# OsVIPP1 Supports Rice Leaf Chloroplast Development and Is Associated with Seed Metabolic Changes and Yield-Related Traits

**DOI:** 10.3390/plants15192946

**Published:** 2026-09-26

**Authors:** Yongli Zhu, Jinghong Zhang, Hongcai Li, Zaihui Zhou, Yongjun Lin, Fei Zhou

**Affiliations:** 1 National Key Laboratory of Crop Genetic Improvement, Huazhong Agricultural University, Wuhan 430070, China; zhuyongli@webmail.hzau.edu.cn (Y.Z.); lihongcai1234@126.com (H.L.); zaihuizhou@mail.hzau.edu.cn (Z.Z.); 2 Key Laboratory of Plant Genetics and Molecular Breeding, Zhoukou Normal University, Zhoukou 466001, China; jinghongzh@outlook.com; 3 Hubei Hongshan Laboratory, Wuhan 430070, China

**Keywords:** OsVIPP1, plastid membrane homeostasis, chloroplast development, transcriptomics, metabolomics, seed metabolism, grain yield

## Abstract

Plastid membrane integrity is essential for plant growth, development, and metabolism, yet the functions of vesicle-inducing protein in plastids 1 (VIPP1) throughout the crop life cycle remain unclear. Here, we characterized *OsVIPP1* in rice using CRISPR/Cas9 knockout, overexpression, and RNA-interference lines, together with ultrastructural, transcriptomic, metabolomic, and targeted biochemical analyses. *osvipp1* knockout seedlings exhibited an albino-lethal phenotype, disorganized thylakoid membranes, disrupted intermembrane connections, and altered expression of chloroplast-encoded genes, supporting an essential role for *OsVIPP1* in plastid membrane development. Both overexpression and RNA interference delayed seed germination, reduced plant height, and impaired yield-related traits. Integrated transcriptomic and untargeted metabolomic analyses associated altered *OsVIPP1* expression with lipid, carbon, and phytohormone-related pathways. In overexpression seeds, abscisic acid content increased to 1.95 times the wild-type level, accompanied by the accumulation of several fatty acids and soluble sugars, whereas abscisic acid and most fatty acids decreased in RNA-interference seeds. These findings indicate that OsVIPP1 supports thylakoid spatial organization in leaves, whereas altered *OsVIPP1* expression is associated with metabolic changes in mature seeds and yield-related phenotypes.

## 1. Introduction

Rice is one of the most important food crops worldwide. Its yield formation relies on the precise coordination of leaf photosynthetic carbon assimilation and seed storage accumulation. As the core organelle for photosynthesis, chloroplasts maintain the structural integrity of the thylakoid membrane system to ensure efficient light harvesting, electron transport, ATP synthesis and carbon fixation [1,2,3], providing continuous carbon sources for grain development and yield formation. In addition to photosynthetic leaves, plastids in mature seeds are involved in lipid metabolism, hormone-related processes and carbon allocation, and their membrane systems may contribute to these processes. Disruption of thylakoid architecture leads to leaf chlorosis, a decline in photosynthetic capacity and suppressed plant growth. Changes in seed plastid homeostasis could influence hormone-related metabolism and storage-substance accumulation (e.g., abscisic acid (ABA)). Nevertheless, key regulators that simultaneously maintain the stability of photosynthetic membranes in leaves and metabolic plastid membranes in seeds, and coordinately govern crop yield formation, remain largely unclear. Therefore, the identification and functional characterization of genes dual-regulating leaf photosynthetic stability and seed metabolic homeostasis are of great significance for elucidating rice yield regulatory mechanisms and facilitating molecular breeding.

Vesicle-inducing protein in plastids 1 (VIPP1) and its prokaryotic homolog phage shock protein A (PspA) are conserved membrane remodeling proteins that have attracted extensive attention in recent years [4]. Prokaryotic PspA acts as a core effector of the Psp stress response system and plays vital roles in maintaining plasma membrane integrity and protecting proton motive force [5]. VIPP1 shares high structural homology with PspA. Both proteins lack typical transmembrane domains but can bind membranes and assemble into large homo-oligomeric ring complexes exceeding 1 MDa [6,7]. Recent structural studies have classified the VIPP1/PspA family into the ESCRT-III superfamily [8]. Cryo-electron microscopy analysis of cyanobacterial VIPP1 revealed membrane curvature-dependent structural transitions from carpet-like sheets to ring and rod assemblies, in which the α0 helix serves as an essential membrane-anchoring domain for membrane tubulation [9]. In the dicot model plant *Arabidopsis thaliana*, null mutation of *AtVIPP1* blocks thylakoid formation and causes embryonic or seedling lethality [10]. Further studies demonstrated that *atvipp1* mutant chloroplasts exhibit balloon-like swelling due to envelope osmotic stress, indicating that VIPP1 is also essential for chloroplast envelope stability [11,12]. Collectively, VIPP1 plays an indispensable and conserved role in maintaining photosynthetic membrane homeostasis.

Chloroplasts function not only as photosynthetic factories but also as central hubs for de novo lipid synthesis, hormone precursor production (e.g., carotenoids for ABA biosynthesis), and carbon allocation [13,14,15]. Plastid lipid metabolism is also closely associated with hormone-related signaling processes, including ABA-responsive pathways [16]. Given the established role of VIPP1 in maintaining plastid membrane homeostasis, these observations raise the possibility that altered OsVIPP1 expression may be associated with changes in lipid- and hormone-related metabolism. Moreover, chloroplast retrograde signaling mediates nuclear transcriptional reprogramming, coordinating sugar metabolism and energy homeostasis at the cellular level [17,18,19]. Importantly, such regulatory mechanisms are not restricted to photosynthetic tissues. Although seed plastids lack photosynthetic activity, their intact membrane systems support lipid metabolism, hormone conversion and carbon redistribution, and are critical for grain filling and seed germination performance. These lines of evidence suggest that VIPP1-mediated membrane homeostasis may extend beyond vegetative photosynthetic regulation, and broadly modulate seed development and germination via reshaping lipid and hormone networks. However, direct experimental evidence supporting this hypothesis is still lacking in cereal crops.

Current knowledge of VIPP1 functions is predominantly derived from prokaryotes and vegetative tissues of dicot plants. The systematic biological functions of VIPP1 throughout the life cycle of monocot crops remain poorly understood. Whether rice OsVIPP1 conserves chloroplast developmental functions similar to *Arabidopsis AtVIPP1*, and whether it additionally regulates seed metabolism and yield formation, remain unresolved questions. Given the distinct differences in chloroplast development, membrane composition and seed developmental patterns between monocot rice and dicot *Arabidopsis*, it is inappropriate to infer rice VIPP1 functions simply based on dicot models. In particular, the potential roles of OsVIPP1 in reproductive growth, seed development and germination have not been explored.

In view of the above research gaps, the present study systematically explores the dual functions of rice *OsVIPP1* in regulating chloroplast development, thylakoid organization and seed metabolism. We generated *osvipp1* knockout mutants via CRISPR/Cas9 editing to verify its essential role in chloroplast development in vegetative tissues. Combined with OE-OsVIPP1 overexpression and RNAi-OsVIPP1 knockdown lines, we further characterized the effects of altered *OsVIPP1* expression on seed metabolism, germination traits and yield components. Multiple experimental approaches were integrated, including chloroplast ultrastructural observation, photosynthetic gene transcription analysis, seed germination evaluation, field agronomic trait investigation, transcriptome and untargeted metabolome profiling, and targeted quantification of endogenous hormones, fatty acids and soluble sugars. This study comprehensively elucidates the multifaceted roles of OsVIPP1 during rice vegetative and reproductive development. Our findings fill the research gap of VIPP1 function across the whole life cycle of monocot crops, and reveal a novel regulatory mechanism by which OsVIPP1 coordinates leaf photosynthesis and seed metabolic homeostasis to govern rice growth and yield formation, providing valuable gene resources and theoretical basis for rice molecular breeding.

## 2. Results

### 2.1. The Osvipp1 Mutant Exhibits the Albino-Lethal Phenotype, Confirming That OsVIPP1 Is an Essential Gene for Chloroplast Development

**Figure 1:**
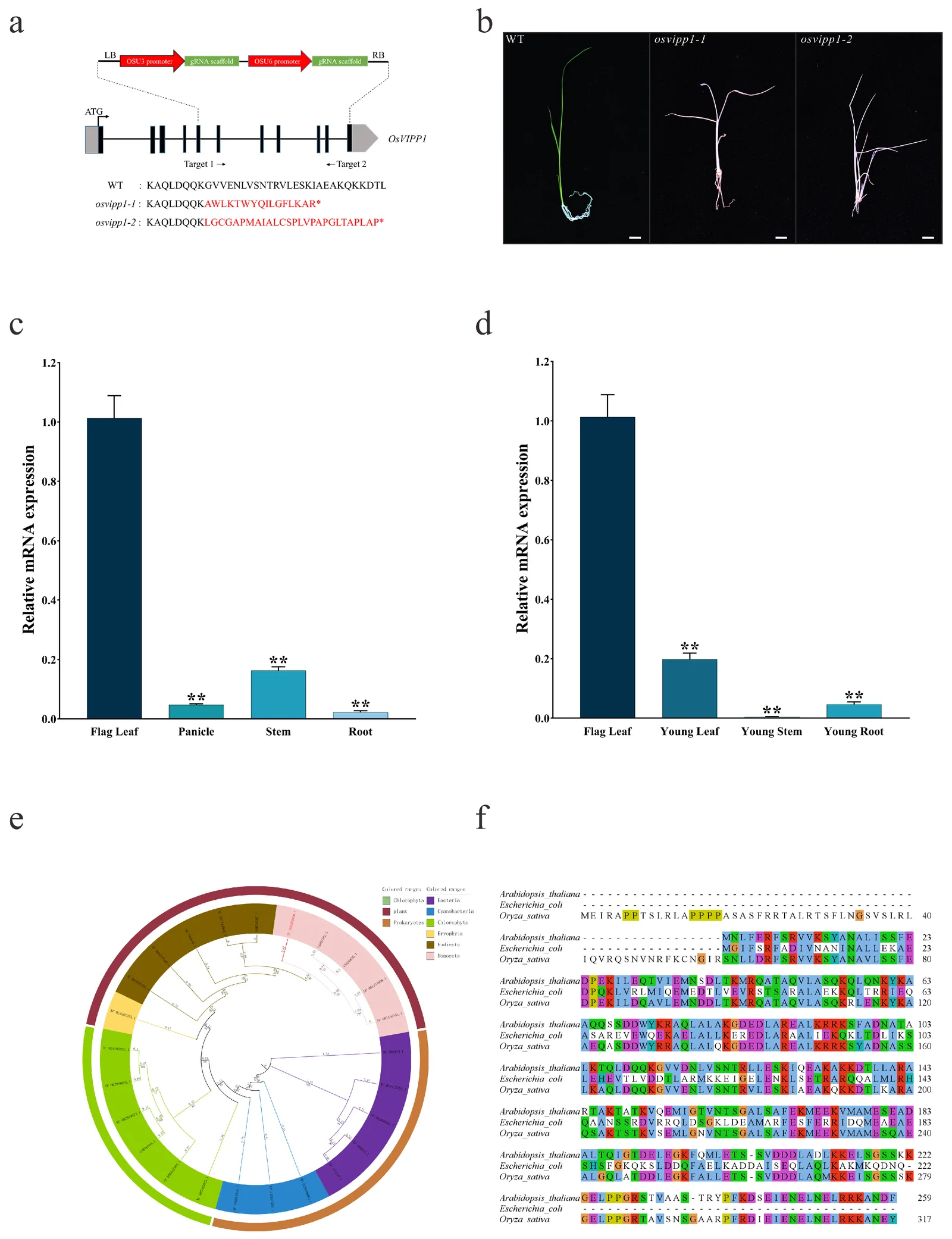
Gene editing, expression pattern and evolutionary analysis of *OsVIPP1*. (**a**) CRISPR/Cas9 target-site design and sequence analysis of mutant alleles of *OsVIPP1*. Both *osvipp1-1* and *osvipp1-2* carried frameshift mutations at Target 1, which resulted in premature termination of protein translation. * represents the stop codon. (**b**) Seedling phenotype of *osvipp1* knockout mutant. Scale bar = 2 cm. (**c**) Relative expression levels of *OsVIPP1* in flag leaves, panicles, stems and roots at heading stage. ** indicates *p* < 0.01. (**d**) Relative expression levels of *OsVIPP1* in young leaves, young stems and young roots of seedlings germinated for approximately 20 d. ** indicates *p* < 0.01. (**e**) Phylogenetic tree of OsVIPP1 and its homologous proteins. (**f**) Multiple-sequence alignment of OsVIPP1, AtVIPP1 and PspA. Data are shown as mean ± SD, *n* = 3. One-way ANOVA was used for statistical analysis.

During the screening of potential OsATP4-interacting proteins, a mutant with severe chloroplast-development defects was isolated. This mutant displayed albino leaves and dwarf stature, and died after 3 weeks of growth on medium supplemented with 2% sucrose. Sanger sequencing revealed a nucleotide deletion near the editing target site, resulting in a frameshift mutation and premature termination of protein translation (Figure 1a). This mutant was designated *osvipp1*. Homozygous *osvipp1* seedlings showed an albino-lethal phenotype at the seedling stage (Figure 1b). Using the modified pCAMBIA1300 vector, we separately introduced a genomic fragment containing the full-length *OsVIPP1* gene together with approximately 2000 bp of its 5′ upstream and 3′ downstream flanking sequences into HPT-negative, complete-knockout calli derived from two independent *osvipp1* mutant families. Because the calli from the two families were cultured and subjected to Agrobacterium-mediated transformation separately, the experiment generated two independent family-level complementation events, yielding a total of 59 green regenerated plantlets. By contrast, all plantlets regenerated from the non-complemented control calli were albino. These results support the conclusion that the albino-lethal phenotype was associated with loss of OsVIPP1 function.

qRT-PCR results indicated that *OsVIPP1* was highly expressed in mature rice tissues, with the highest transcript abundance in leaves, followed by stems and panicles (Figure 1c). Its overall expression was relatively low in seedling tissues, whereas transcript level in seedling roots was higher than that in mature roots (Figure 1d). These results suggest that *OsVIPP1* exhibits obvious tissue-specific and development-dependent expression patterns. Phylogenetic tree and multiple-sequence alignment revealed that VIPP1 proteins are relatively conserved among rice, *Arabidopsis thaliana* and *Escherichia coli* (Figure 1e). Sequence divergences mainly occur at the N- and C-termini. Nevertheless, the core domain of VIPP1 maintains continuous sequence correspondence and is highly conserved across species (Figure 1f).

### 2.2. OsVIPP1 Localizes to Chloroplasts, and Its Loss Causes Disorganized Thylakoid Architecture and Perturbed Transcription of Photosynthetic-Related Chloroplast Genes

Subcellular localization assays demonstrated that OsVIPP1 is targeted to chloroplasts (Supplementary Figure S1a). In rice protoplasts, the OsVIPP1-GFP fusion protein exhibited distinct punctate chloroplastic fluorescence signals, which displayed prominent dynamic features upon prolonged protoplast incubation (Supplementary Figure S2). Transmission electron microscopy further revealed that compared with WT, chloroplasts of the *osvipp1* mutant displayed loosely arranged thylakoid membranes, disrupted inter-lamellar connections and local structural disorder, resulting in severely compromised ultrastructural integrity. By contrast, thylakoid and grana architecture remained normal in OE-OsVIPP1 overexpression lines (Supplementary Figure S1b). These observations indicate that *OsVIPP1* is indispensable for maintaining the spatial organization of thylakoid membranes in leaf tissues. Loss of *OsVIPP1* disrupts chloroplast membrane architecture.

To determine whether chloroplast structural defects are accompanied by functional transcriptional alterations, qRT-PCR was performed to examine transcript levels of plastid-encoded genes in three-leaf-stage leaves. Genes encoding ATP-synthase subunits, including *atpA*, *atpB*, *atpE* and *atpF*, were significantly down-regulated (Supplementary Figure S3a), suggesting repression of chloroplastic energy-conversion processes. Among NDH-complex genes, *ndhB* was up-regulated, whereas *ndhA*, *ndhC*, *ndhD* and *ndhK* were markedly down-regulated (Supplementary Figure S3b). PSI-associated genes *psaB*, *psaC* and *psaJ* showed increased transcript abundance. PSII-related genes exhibited divergent expression patterns: *psbC*, *psbE*, *psbF* and *psbJ* were significantly up-regulated, with the strongest induction observed for *psaJ* and *psbJ*. Meanwhile, *psbA*, *psbB*, *psbD* and *psbH* were repressed, especially *psbD* and *psbH* (Supplementary Figure S3c). Most chloroplast ribosomal-protein genes, such as *rps3*, *rps8*, *rps12*, *rps19*, *rpl20* and *rpl32*, accumulated to higher levels (Supplementary Figure S3e,f). Collectively, loss of *OsVIPP1* not only induces structural defects in thylakoid membranes, but also triggers transcriptional perturbation of chloroplast genes associated with the photosynthetic apparatus and energy-conversion pathways.

### 2.3. Effects of Altered OsVIPP1 Expression on Rice Phenotype, Seed Germination, and Yield-Related Traits

**Figure 2:**
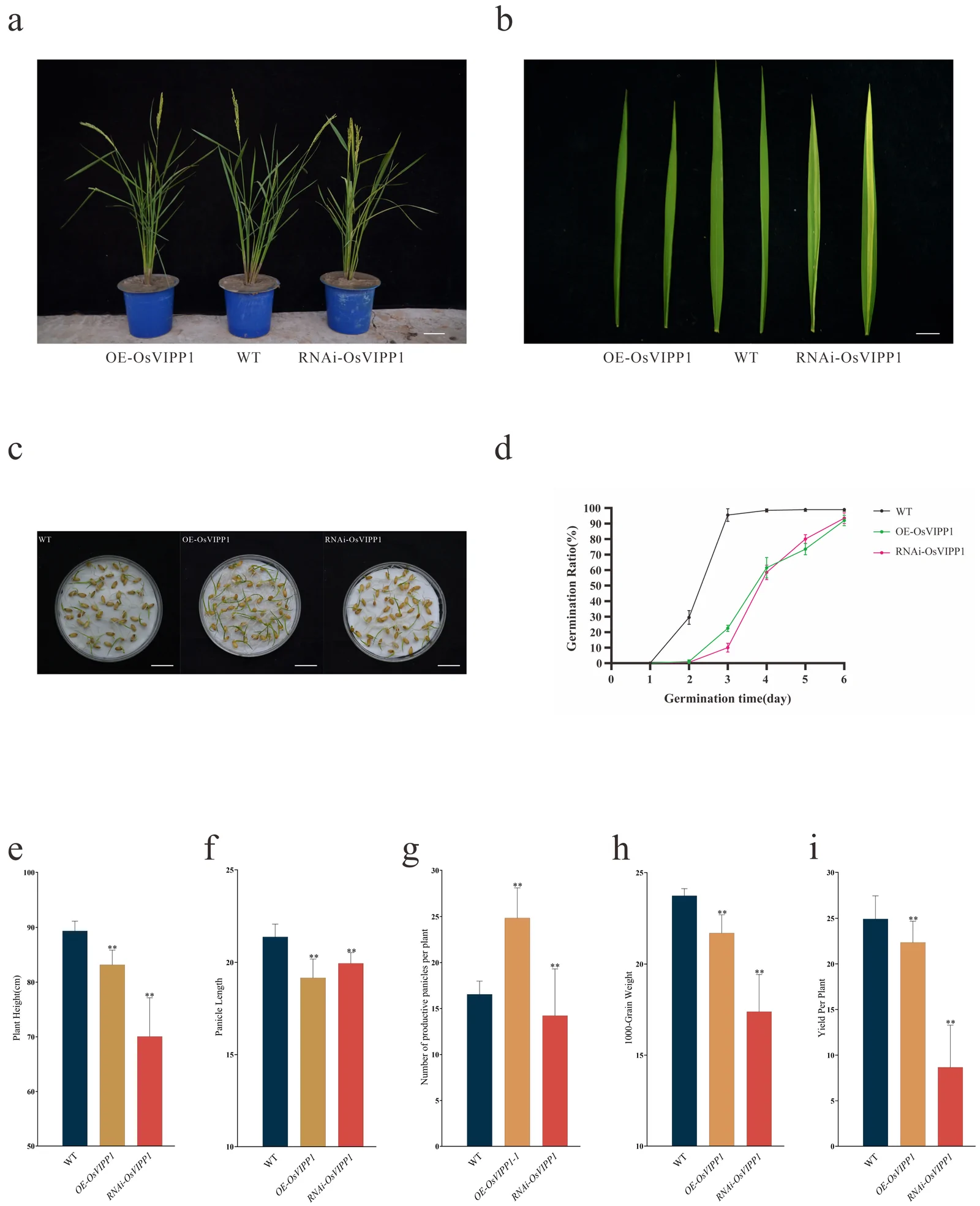
Phenotypes and seed-germination characteristics of OE-OsVIPP1 and RNAi-OsVIPP1 plants. (**a**) Mature-stage plant phenotypes of OE-OsVIPP1, WT and RNAi-OsVIPP1. Scale bar = 10 cm. (**b**) Leaf phenotypes: The two leaves on the left are from OE-OsVIPP1, the two in the middle from WT, and the two on the right from RNAi-OsVIPP1. Scale bar = 2 cm. (**c**) Seed-germination phenotypes shown at day 3 after sowing. (**d**) Time-course curve of seed germination rate. For panels (**c**,**d**), *n* = 3 represents three independent Petri dishes, each treated as one biological replicate. (**e**–**i**) Statistical results of plant height, panicle length, effective panicle number, 1000-grain weight and grain yield per plant. ** indicates *p* < 0.01. Data are shown as mean ± SD, *n* = 3. One-way ANOVA was used for statistical analysis.

To further explore the regulatory roles of *OsVIPP1* in rice growth and development throughout the whole life cycle, the field phenotypes at the heading stage and seed-germination characteristics of OE-OsVIPP1 and RNAi-OsVIPP1 lines were systematically analyzed. Compared with WT, both OE-OsVIPP1 and RNAi-OsVIPP1 plants exhibited significantly shortened flag leaves. In particular, RNAi lines displayed a mild albino phenotype similar to the *osvipp1* knockout mutant, with white streaks appearing on partial leaf regions (Figure 2a,b). These abnormal morphological features indicate defects in chloroplast-related development and pigment-associated processes in vegetative tissues.

Seed germination assays showed that the final germination rates of OE-OsVIPP1 and RNAi-OsVIPP1 seeds were comparable to those of WT. However, seed germination initiation was delayed, with increased T_50_ and MGT values (Supplementary Table S8) and reduced germination speed in both transgenic lines (Figure 2c,d). Quantitative analysis confirmed this delay. The T_50_ values were 2.30 ± 0.04 days in WT, 3.61 ± 0.05 days in OE-OsVIPP1, and 3.77 ± 0.11 days in RNAi-OsVIPP1. Consistently, the MGT values were 2.74 ± 0.03, 4.28 ± 0.03, and 4.40 ± 0.07 days, respectively (Supplementary Table S8). Since seed germination primarily depends on the mobilization of internal reserved substances rather than newly synthesized photosynthates from leaves, these results suggest that OsVIPP1 participates in the establishment of germination-related metabolic programs during seed maturation. Accordingly, the function of OsVIPP1 is not limited to chloroplast development and chloroplast-related processes in leaf tissues.

Statistical analysis of agronomic traits revealed that plant height, panicle length, 1000-grain weight, and single-plant yield were decreased in both OE-OsVIPP1 and RNAi-OsVIPP1 lines. Interestingly, the effective panicle number was increased by approximately eight on average in OE-OsVIPP1 plants (Figure 2e–i). Although most yield-related traits showed consistent reduction trends in overexpression and RNAi lines, the underlying causes of yield impairment differed between the two genotypes. The mild albino phenotype of RNAi-OsVIPP1 plants suggests that their yield reduction may be associated with defects in chloroplast development and leaf structural integrity. Because direct photosynthetic measurements were not performed, we do not attribute this phenotype to reduced carbon assimilation. In contrast, OE-OsVIPP1 plants exhibited normal thylakoid ultrastructure, implying that their yield reduction may be primarily caused by impaired grain development and insufficient substance accumulation during seed maturation. Furthermore, GUS staining showed strong OsVIPP1 accumulation at the leaf pulvinus of stems (Supplementary Figure S4), which may account for the increased effective panicle number in OsVIPP1 overexpression plants.

### 2.4. Altered OsVIPP1 Expression Is Associated with Transcriptional Changes in Fatty Acid, Hormone and Carbon Metabolism in Seeds

To dissect the molecular responses triggered by altered OsVIPP1 expression in mature seeds, transcriptome analysis was performed on mature seeds of WT, OE-OsVIPP1 and RNAi-OsVIPP1. Principal-component analysis (PCA) revealed clear separation among three genotypes at the transcriptomic level, with PC1 and PC2 explaining 38.51% and 23.43% of total variance, respectively (Supplementary Figure S5a). We identified up-regulated genes in OE-OsVIPP1 relative to WT and down-regulated genes in RNAi-OsVIPP1 relative to WT, and 319 overlapping genes were obtained from these two gene sets (Supplementary Figure S5b).

**Figure 3:**
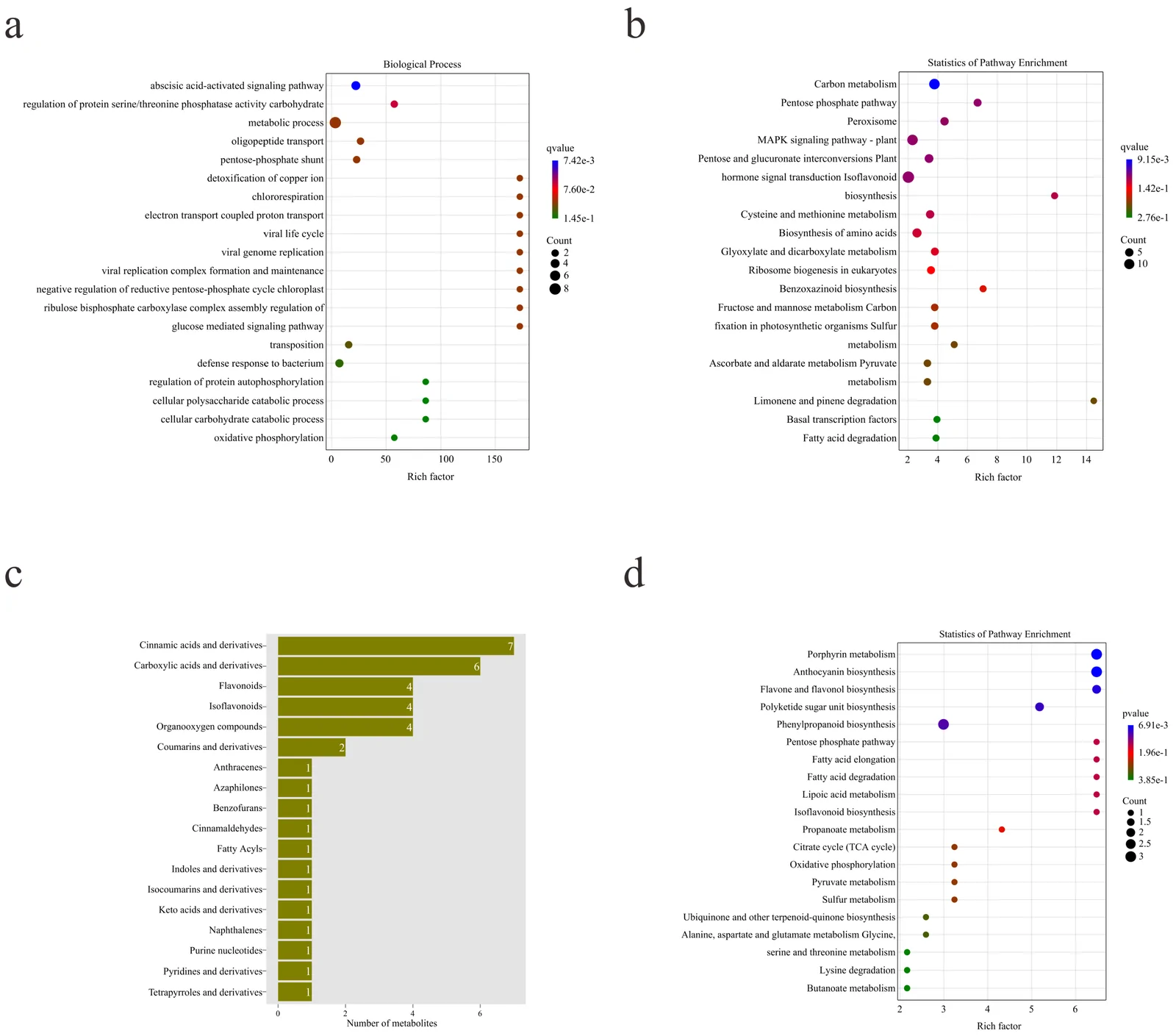
Transcriptome and metabolome analyses of WT, OE-OsVIPP1 and RNAi-OsVIPP1 materials. Three independent biological replicates were used for each genotype in both analyses. (**a**) GO enrichment analysis of co-differentially expressed genes. (**b**) KEGG pathway enrichment analysis of co-differentially expressed genes. (**c**) Cluster analysis of co-differentially accumulated metabolites. (**d**) KEGG pathway enrichment analysis of co-differentially accumulated metabolites.

GO enrichment analysis demonstrated that these overlapping genes were mainly enriched in biological processes including abscisic acid signaling pathway, carbohydrate metabolism, pentose-phosphate pathway, chloroplast respiration, electron-transport-coupled process, cellular polysaccharide and carbohydrate catabolic process, among which the abscisic acid signaling pathway showed the most prominent enrichment (Figure 3a). KEGG enrichment further indicated that these genes were predominantly involved in carbon metabolism, pentose-phosphate pathway, plant MAPK signaling pathway, plant hormone signal transduction, carbon fixation in photosynthetic organisms, and fatty-acid degradation (Figure 3b). Collectively, in mature seeds that lack photosynthetic activity, changes in *OsVIPP1* expression are tightly linked to transcriptional regulation of genes related to carbon metabolism, hormone response and lipid metabolism, suggesting that OsVIPP1 also plays an essential role in metabolic regulation in seeds.

### 2.5. Effects of Altered OsVIPP1 Expression on Seed Metabolome

To further elucidate the regulatory effects of OsVIPP1 on seed metabolism, untargeted metabolomic profiling was performed using mature seeds of WT, OE-OsVIPP1 and RNAi-OsVIPP1. PCA revealed distinct metabolic separation among the three genotypes, with PC1 and PC2 accounting for 33.0% and 23.5% of the total variance, respectively (Supplementary Figure S5c). A total of 271 overlapping differential metabolites were identified by comparing the up-accumulated metabolites in OE-OsVIPP1 versus WT and the down-accumulated metabolites in RNAi-OsVIPP1 versus WT (Supplementary Figure S5d). Classification statistics showed that these differential metabolites mainly belonged to cinnamic acid and its derivatives, carboxylic acid derivatives, flavonoids, isoflavonoids, and oxygenated organic compounds (Figure 3c). KEGG enrichment analysis indicated that these metabolites were primarily enriched in porphyrin metabolism, anthocyanin biosynthesis, flavonoid and flavonol biosynthesis, phenylpropanoid biosynthesis, fatty acid metabolism, the pentose phosphate pathway, the TCA cycle, and oxidative phosphorylation (Figure 3d). Collectively, altered *OsVIPP1* expression was associated with metabolic changes during seed development involving secondary metabolism, energy metabolism and lipid metabolism.

### 2.6. Integrated Transcriptome and Metabolome Analysis Identifies Shared Pathways Associated with OsVIPP1-Regulated Seed Metabolism

**Figure 4:**
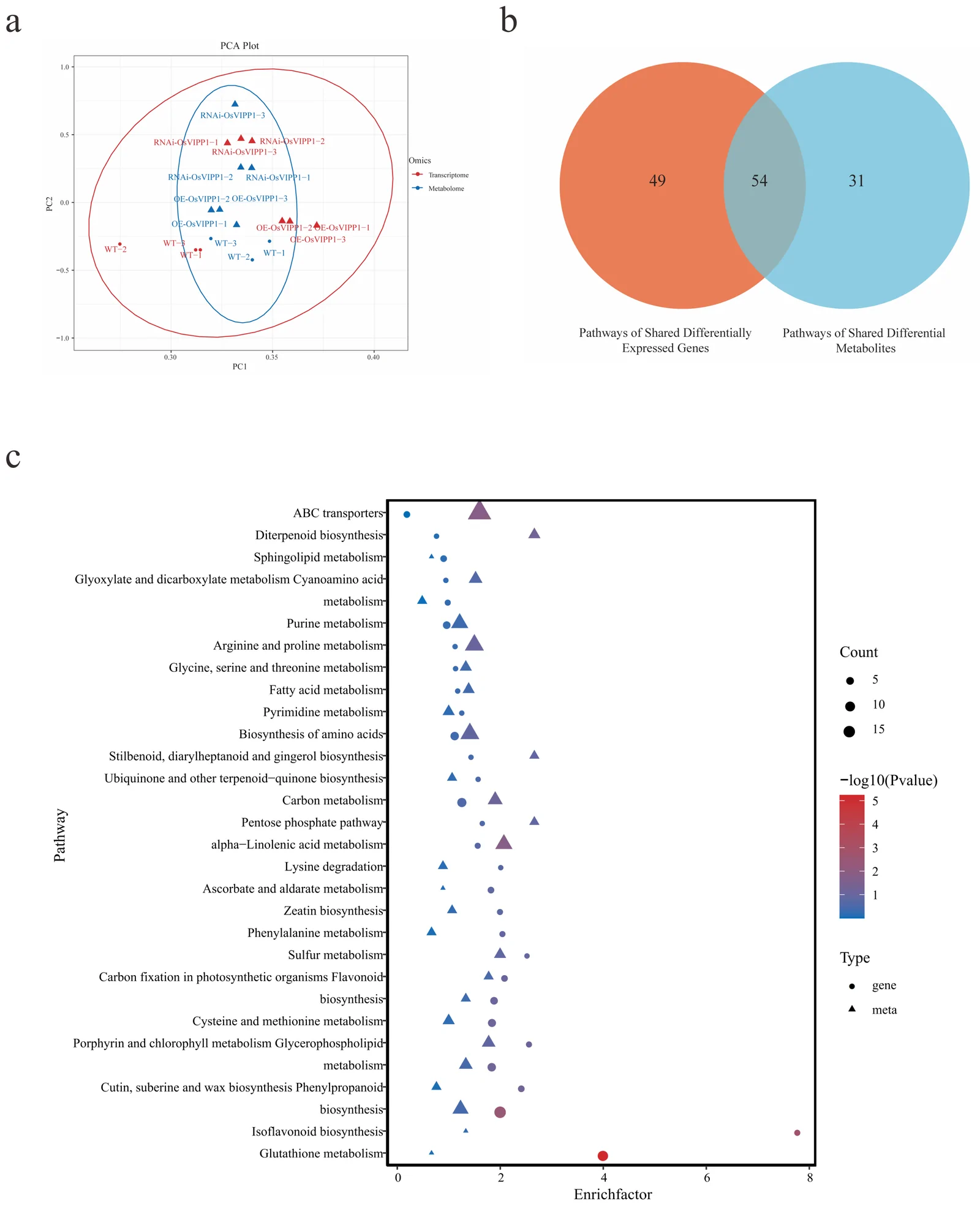
Integrated transcriptome–metabolome analysis of WT, OE-OsVIPP1 and RNAi-OsVIPP1 lines. The integrated analysis was based on transcriptomic and metabolomic datasets generated from three independent biological replicates per genotype. (**a**) Combined PCA of transcriptomic and metabolomic datasets. (**b**) Venn diagram showing overlapping enriched pathways derived from transcriptome and metabolome. (**c**) Integrated analysis of shared enriched pathways between transcriptome and metabolome. Circles represent genes, and triangles represent metabolites. Symbol size indicates the number of genes or metabolites, and color denotes −log_10_(*p*-value).

Integrated multi-omics analysis showed that transcriptomic and metabolomic samples of WT, OE-OsVIPP1 and RNAi-OsVIPP1 were clearly distinguished in two-dimensional clustering (Figure 4a). Biological replicates of each genotype clustered closely with good intra-group consistency, while distinct inter-group separation was observed among different genotypes. These results indicated that altered *OsVIPP1* expression induces stable and specific molecular variations at both transcriptional and metabolic levels. Pathway intersection analysis identified 49 unique enriched pathways at the transcript level and 31 unique pathways at the metabolite level, with 54 overlapping pathways shared by transcriptome and metabolome datasets (Figure 4b). KEGG integration analysis revealed that the overlapping pathways were mainly involved in carbon metabolism, pentose phosphate pathway, fatty acid metabolism, α-linolenic acid metabolism, amino acid biosynthesis, purine and pyrimidine metabolism, cysteine and methionine metabolism, porphyrin and chlorophyll metabolism, and glycerophospholipid metabolism. Among them, glutathione metabolism, isoflavonoid biosynthesis and phenylpropanoid biosynthesis exhibited higher transcriptional significance, whereas lipid-related pathways such as α-linolenic acid metabolism were more prominent at the metabolic level (Figure 4c). Collectively, perturbation of OsVIPP1 expression was associated with changes at both the transcriptomic and metabolomic levels. The overlapping pathways mainly involved redox homeostasis, lipid metabolism, carbon metabolism, secondary metabolism and hormone-related metabolism, indicating biological processes associated with OsVIPP1-regulated seed metabolism.

### 2.7. Altered OsVIPP1 Expression Affects the Accumulation of Hormones, Fatty Acids, and Soluble Sugars in Seeds

**Figure 5:**
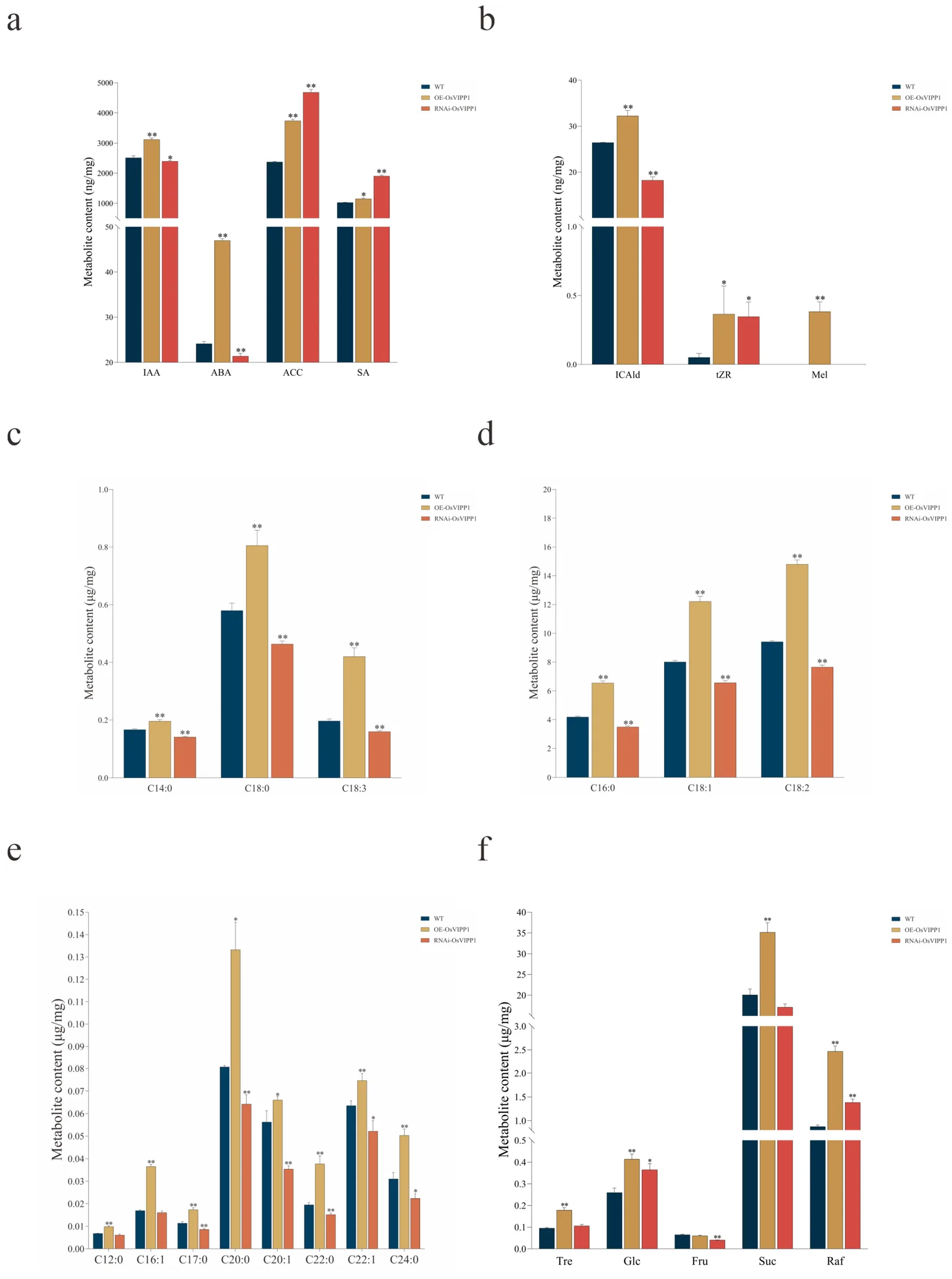
Quantification of plant hormones, fatty acids and soluble sugars in seeds of WT, OE-OsVIPP1 and RNAi-OsVIPP1 lines. ** indicates *p* < 0.01; * indicates *p* < 0.05. (**a**) Contents of hormone-related metabolites IAA, ABA, ACC and SA. (**b**) Quantification of cytokinins and other hormone-related metabolites ICAld, tZR and Mel. (**c**) Contents of fatty acids C14:0, C18:0 and C18:3. (**d**) Contents of fatty acids C16:0, C18:1 and C18:2. (**e**) Contents of fatty acids C12:0, C16:1, C17:0, C20:0, C20:1, C22:0, C22:1 and C24:0. (**f**) Quantification of soluble sugars Tre (trehalose), Glc (glucose), Fru (fructose), Suc (sucrose), and Raf (raffinose). Panels (**a**,**b**,**f**) use broken y-axes to accommodate data spanning multiple orders of magnitude. Data are shown as mean ± SD, *n* = 3. One-way ANOVA was used for statistical analysis.

Targeted quantitative analysis was performed to further characterize the effects of altered *OsVIPP1* expression on seed metabolites. OsVIPP1 significantly altered the accumulation of multiple plant hormones, including ABA (Figure 5a,b). The ABA content was 24.14 ± 0.49 ng/g in WT seeds, increased to 46.95 ± 0.43 ng/g in OE-OsVIPP1 seeds (approximately 1.95-fold of the WT level), and decreased to 21.38 ± 0.61 ng/g in RNAi-OsVIPP1 seeds. Other hormone-related metabolites, including IAA, ACC, SA, ICAld, and tZR, were also differentially accumulated in response to altered *OsVIPP1* expression. Notably, OE-OsVIPP1 and RNAi-OsVIPP1 seeds exhibited opposite ABA variation patterns, whereas both genotypes displayed delayed seed germination. This contradictory phenomenon implies that overexpression and RNAi lines may achieve consistent germination inhibition through distinct metabolic regulatory pathways.

Fatty acid quantification showed that most fatty acid components, including C14:0, C16:0, C18:0, C18:1, C18:2, C18:3, C20:0, C20:1, C22:0, C22:1, and C24:0, were significantly elevated in OE-OsVIPP1 seeds (Figure 5c–e). In contrast, RNAi-OsVIPP1 seeds generally contained lower fatty acid levels than WT, showing opposite metabolic responses between the overexpression and RNAi lines. Soluble sugar analysis revealed that OE-OsVIPP1 seeds accumulated higher amounts of trehalose, glucose, sucrose, and raffinose. In RNAi-OsVIPP1 seeds, fructose and sucrose contents were decreased, whereas glucose and raffinose remained higher than WT levels (Figure 5f). Collectively, altered *OsVIPP1* expression was associated with changes in ABA-related hormone metabolism, fatty acid accumulation and soluble sugar homeostasis in rice seeds. Opposite metabolic alterations were frequently observed between OE-OsVIPP1 and RNAi-OsVIPP1 lines, indicating bidirectional metabolic responses to OsVIPP1 perturbation.

## 3. Discussion

### 3.1. OsVIPP1 Plays Dual Roles in Thylakoid Spatial Organization and Photosynthetic Performance Maintenance in Rice Leaves

Ordered stacking and inter-membrane connections of thylakoids constitute the structural foundation for compartmentalized assembly and functional coordination of photosynthetic complexes [20]. In *Arabidopsis thaliana* and *Chlamydomonas reinhardtii*, loss-of-function or knockdown of VIPP1 severely impairs thylakoid biogenesis and drastically reduces the abundance of photosynthetic membranes, supporting its conserved and indispensable role in photosynthetic membrane biogenesis [10,21]. In the present study, the rice *osvipp1* knockout mutant exhibited partially divergent phenotypes compared with those reported in dicot models. Although basic thylakoid membranes were still detectable in mutant chloroplasts, thylakoid lamellae were loosely arranged with greatly diminished inter-membrane connections. This observation indicates that OsVIPP1 is primarily required for continuity and higher-order spatial organization of the thylakoid membrane network, rather than for de novo membrane-lipid biosynthesis or total membrane biogenesis. Such species-specific phenotypes imply that rice chloroplast membrane development possesses partial functional redundancy or compensatory mechanisms, which permit basal membrane synthesis in the absence of OsVIPP1 yet fail to restore proper higher-order spatial architecture. Exogenous carbon supply via sucrose-rich medium failed to rescue mutant lethality, consistent with previous findings in *Arabidopsis atvipp1* mutants [10]. This further supports the view that the physical integrity of thylakoid membrane connections is essential for normal chloroplast development and seedling survival, and this structural requirement cannot be bypassed by external carbon sources.

Notably, chloroplast-encoded genes in *osvipp1* displayed complex transcriptional reprogramming. Genes encoding ATP-synthase subunits (*atpA*, *atpB*, *atpE*, *atpF*, etc.) and NDH-complex subunits (*ndhA*, *ndhC*, *ndhD*, *ndhK*) were generally down-regulated, whereas several PSI- and PSII-related genes were up-regulated. This phenomenon of “co-existing compensatory expression and functional impairment” provides novel experimental evidence from monocot crops for ongoing debates concerning VIPP1 function. Early studies in *Arabidopsis* proposed that VIPP1 facilitates thylakoid formation by mediating intraplastidic vesicle trafficking; its deficiency eliminates vesicles, triggers thylakoid degradation and leads to seedling lethality [10]. Nevertheless, this hypothesis has been challenged by subsequent work. VIPP1 knockdown in *Chlamydomonas* did not cause severe ultrastructural collapse of thylakoids but reduced the accumulation of multiple photosynthetic complexes and ATP synthase by 14–20% [21]. In cyanobacteria, the decline in photosynthetic activity upon VIPP1 depletion preceded obvious thylakoid deterioration [22]. Further studies in *Arabidopsis* revealed chloroplast balloon-like envelope swelling in *atvipp1* mutants, whereas grana stacking remained relatively intact [11,12]. However, these conclusions were mainly derived from cyanobacteria, algae and *Arabidopsis*, and direct evidence in rice is missing. Our results support an integrated model featuring structure-dependent and structure-independent regulatory modes for VIPP1-dependent photosynthetic maintenance. On one hand, OsVIPP1 guarantees proper anchoring and functional coordination of photosynthetic complexes by preserving spatial organization of thylakoid membranes (structure-dependent pathway). On the other hand, it may modulate photosystem activity by influencing chloroplast gene transcription or photosynthetic-complex assembly efficiency (structure-independent pathway). The combinatorial effects of these two pathways explain why systemic transcriptional perturbation of photosynthetic genes occurs even before complete disintegration of thylakoid architecture in *osvipp1* mutants.

### 3.2. OsVIPP1 Contributes to Yield Formation by Coordinately Modulating Leaf Carbon Supply and Seed Grain Metabolism

Proper organization of thylakoid membranes provides structural support for efficient photosynthetic electron transport and ATP synthesis, and photosynthetic capacity directly governs biomass accumulation and yield performance in crops [20]. Accordingly, defects in thylakoid architecture are frequently reflected in plant growth and yield-related traits. Nevertheless, final yield is determined not only by photosynthetic carbon supply from source leaves but also by the capacity of grains to import and utilize assimilates for grain filling. Complete loss-of-function of VIPP1 causes seedling lethality in *Arabidopsis* [10] and tomato [23], preventing mutants from entering reproductive growth. Thus, direct genetic evidence linking VIPP1 to crop yield formation remains scarce. In the present study, rice overexpression and RNA-interference lines were used to overcome this experimental limitation. Although *OsVIPP1* expression was altered in opposite directions in overexpression and RNA-interference plants, both genotypes exhibited shortened flag leaves, reduced plant height, decreased panicle length, lower 1000-grain weight and reduced yield per plant. Further analyses revealed that yield depression arose from distinct underlying pathways in the two types of transgenic lines.

RNA-interference lines displayed prominent phenotypic alterations, including mild leaf albino streaks, shortened flag leaves, reduced 1000-grain weight and decreased yield per plant. Their yield reduction may be associated with defects in chloroplast development and leaf structural integrity. By contrast, overexpression lines possessed thylakoid ultrastructure comparable to wild-type plants, so their yield decline could not be explained by severe membrane damage as observed in knockout mutants. Intriguingly, overexpression lines showed a paradoxical “more panicles but fewer grains” phenotype: the number of effective panicles increased by approximately eight on average, whereas 1000-grain weight and per-plant yield decreased. This indicates that *OsVIPP1* overexpression promotes tillering and productive panicle formation without concurrent improvement in single-panicle productivity. The beneficial effect of increased panicle number was offset by defects occurring at the grain-filling stage. Therefore, evaluating the yield-regulatory effect of OsVIPP1 cannot rely merely on tiller or panicle count; coordinated improvement among multiple yield-component traits should be considered.

The “more panicles but fewer grains” phenotype of overexpression lines is most likely caused by impaired grain-filling capacity rather than by the leaf structural defects observed in RNA-interference lines. Targeted metabolic measurements revealed significant accumulation of ABA, diverse fatty acids and soluble sugars in overexpressing seeds. Such metabolic status is tightly associated with compromised grain filling, providing a metabolic explanation for reduced 1000-grain weight. Hence, yield loss in overexpression lines appears to originate largely from intrinsic disturbances in seed development and metabolism, which differs from the chloroplast-development-associated phenotype observed in RNA-interference lines. Because the RNA-interference and overexpression lines showed distinct phenotypic and metabolic changes that converged on reduced grain yield, we propose a working model in which RNA-interference lines may exhibit source-limited yield reduction associated with chloroplast-development and leaf-structure defects, whereas overexpression lines may exhibit sink-limited yield reduction associated with altered seed development and metabolism. This distinction remains to be tested.

### 3.3. Contrasting Seed Metabolic Responses Associated with OsVIPP1 Perturbation

Seed germination is coordinately controlled by phytohormones and endogenous metabolites. ABA acts as a major repressor of seed germination [24]. Soluble sugars serve not only as energy substrates but also as signaling molecules that trigger metabolic re-activation during early germination [25]. Nevertheless, whether and how seed plastid membrane homeostasis is related to germination control through hormone and metabolite changes remains unclear. In this study, both overexpression and RNA-interference lines exhibited delayed seed germination, whereas their final germination rates were comparable to wild-type seeds. This indicates that perturbed *OsVIPP1* expression primarily delays germination speed rather than abolishing seed-germination capacity. Further metabolic profiling revealed opposite metabolic signatures in the two transgenic genotypes. Seeds from overexpression lines accumulated higher levels of ABA, fatty acids and most soluble sugars, whereas RNA-interference seeds showed reduced ABA and the majority of fatty acids. Other hormone-related metabolites also changed among the genotypes. IAA increased in OE-OsVIPP1 seeds but showed a modest decrease in RNAi-OsVIPP1 seeds, whereas ACC and SA increased in both transgenic genotypes. ICAld increased in OE-OsVIPP1 seeds but decreased in RNAi-OsVIPP1 seeds, and tZR increased in both genotypes. Melatonin was mainly increased in OE-OsVIPP1 seeds. Despite the opposite ABA responses, both transgenic genotypes exhibited delayed seed germination, indicating that ABA alone cannot explain the germination phenotype. These results indicate that the shared germination delay is associated with genotype-specific hormonal and metabolic changes, but the present metabolomic data do not establish direct metabolic causality or a resolved hormonal mechanism.

Multi-omics analyses of mature seeds provide network-level insights into this regulatory pattern. In total, 319 shared differentially expressed genes and 271 shared differential metabolites were identified, which were enriched in carbon metabolism, pentose phosphate pathway and fatty acid metabolism—core routes for plastid membrane–lipid biosynthesis and carbon partitioning. Evidence from cyanobacteria indicates that VIPP1 is functionally associated with lipid metabolism, as co-expression of VIPP1 and an acyl-lipid desaturase alters the composition of polyunsaturated fatty acids and MGDG/DGDG galactolipids [26]. This finding supports a possible connection between VIPP1 function and membrane lipid remodeling. However, whether a comparable mechanism operates in rice seed plastids remains unclear. Therefore, the altered fatty-acid abundance observed in rice seeds may reflect secondary changes in plastid lipid homeostasis or carbon allocation, and this possibility requires further experimental validation. Taken together, either increased or decreased *OsVIPP1* expression disturbs plastid membrane homeostasis, which is associated with broad changes in lipid-, hormone- and sugar-related metabolic processes in seeds.

### 3.4. Functional Implications and Breeding Prospects of OsVIPP1

Collectively, our results demonstrate that OsVIPP1 acts as a pleiotropic regulator governing both vegetative and reproductive development in rice. During vegetative growth, OsVIPP1 supports chloroplast development by sustaining the spatial organization of thylakoid membranes. During reproductive growth, altered *OsVIPP1* expression was associated with changes in seed lipid, hormone and soluble sugar metabolism, germination and yield-related traits. A possible contribution of seed plastid membrane homeostasis is proposed, but it is not directly demonstrated here. These regulatory pathways converge on the core function of OsVIPP1 in maintaining plastid membrane homeostasis. Altered *OsVIPP1* expression, including knockout, knockdown and overexpression, may affect the coordination between leaf assimilate supply and grain assimilate accumulation, thereby contributing to yield reduction. These observations suggest a possible connection between VIPP1 function, source-sink coordination and crop yield modulation.

This study has certain limitations and provides clear directions for future research. First, the seedling-lethal phenotype of *osvipp1* null mutants prevents functional characterization during reproductive growth. Inducible or tissue-specific expression systems can be used to achieve spatiotemporal manipulation of OsVIPP1, thereby distinguishing its independent and overlapping roles in leaf chloroplast development and seed metabolism. Second, the transcriptomic and metabolomic data in this study were obtained from mature dry seeds, reflecting metabolic reserves before germination. Dynamic metabolic reprogramming during seed imbibition and germination remains to be explored through time-series multi-omics analysis. In addition, previous studies have shown that algal VIPP2 mediates chloroplast membrane stress-triggered retrograde signaling [27], and the VIPP1-interacting protein VIA1 participates in thylakoid biogenesis [28], implying conserved VIPP1 regulatory networks in plants. Identification of OsVIPP1’s interacting factors and downstream pathways will further elucidate the molecular mechanism linking membrane homeostasis and metabolic reprogramming.

From the perspective of molecular breeding, this study provides valuable theoretical insights. Constitutive overexpression of *OsVIPP1* causes significant yield penalties, indicating that simple upregulation of functional genes cannot always generate favorable agronomic traits, and precise modulation of gene expression is essential. Given the contrasting metabolic and developmental responses associated with altered *OsVIPP1* expression, OsVIPP1 may be more suitable for precise genetic optimization than for constitutive overexpression. Future studies should explore natural allelic variations, promoter haplotypes, and tissue- and development-specific expression patterns of *OsVIPP1* to identify regulatory modes that balance leaf chloroplast development and seed metabolic homeostasis.

## 4. Materials and Methods

### 4.1. Plant Materials and Growth Conditions

Rice cultivar Zhonghua 11 (*Oryza sativa* L. ssp. *japonica* cv. Zhonghua 11, ZH11) was used as the wild-type (WT) background in this study. All mutants, RNA-interference (RNAi) and overexpression lines were generated in the ZH11 background. Field trials were performed at the experimental field of Huazhong Agricultural University (30.4° N, 114.2° E), and all plant materials were grown under identical field management conditions. T_1_ generation materials for loss-of-function phenotypic observation were cultured in vitro. Briefly, seeds were surface-sterilized and sown on 1/2 MS medium, then germinated and grown at 28 °C under a 14 h light/10 h dark photoperiod. T_2_ seeds of the OsVIPP1 overexpression and RNAi lines used for phenotypic characterization were sown together with WT materials in early April 2023 and transplanted to the field at the three-leaf stage in mid-April. Three plots were established in three separate seedling fields, with each plot measuring 6 m in length and 2 m in width. Three independent families were selected for each genotype, with 30 plants per family and 10 plants planted per row. The row spacing was 25 cm and the plant spacing was 15 cm. Pest and disease control was performed once, one month after transplanting.

In this study, each transgenic family represented an independent T_0_ transformation event. The three independent *OsVIPP1* overexpression events were designated OE-OsVIPP1-1, OE-OsVIPP1-2 and OE-OsVIPP1-3, whereas the three independent RNAi events were designated RNAi-OsVIPP1-1, RNAi-OsVIPP1-2 and RNAi-OsVIPP1-3. These families were used in the germination, agronomic-trait, transcriptomic, metabolomic and biochemical analyses described below, and the above identifiers are used consistently throughout the manuscript. Unless otherwise stated, the three independent transgenic families were treated as biological replicates.

### 4.2. Construction of Osvipp1 Knockout, RNAi Knockdown and Overexpression Materials

The *osvipp1* knockout mutant was generated using the CRISPR/Cas9 system. Two pairs of sgRNAs targeting *OsVIPP1* were designed via the CRISPR-P online tool and cloned into the pYLCRISPR/Cas9-MH vector. The recombinant construct was introduced into ZH11 calli via Agrobacterium-mediated transformation to produce T_0_ transgenic plants. T_0_ individuals were subjected to PCR amplification and sequencing genotyping. Homozygous mutants carrying frameshift mutations predicted to cause premature protein termination were identified and used for subsequent analyses. Primers are listed in Supplementary Table S1. Information on the *OsVIPP1* sgRNA target sites is provided in Supplementary Table S2.

For genetic complementation of the *osvipp1* mutant, a genomic fragment containing the full-length OsVIPP1 gene together with 2000 bp of upstream and downstream flanking sequences was amplified and cloned into the modified pCAMBIA1300 vector. The resulting recombinant construct was introduced into calli derived from homozygous *osvipp1* mutants by Agrobacterium-mediated transformation [29]. *HPT* was used as the selectable marker for transgenic selection. Regenerated plantlets were subjected to genotyping and phenotypic characterization. Primers used for vector construction and genotyping are listed in Supplementary Table S1.

RNAi-OsVIPP1 lines were constructed based on a double-stranded RNA-mediated silencing strategy. The specific fragment of OsVIPP1 was amplified from ZH11 cDNA using primers RNAi-OsVIPP1-F and RNAi-OsVIPP1-R. The amplified fragment was cloned into the pCR8 vector and further recombined into the DS355 vector by LR recombination to generate the RNAi-OsVIPP1 expression construct. The recombinant vector was transformed into ZH11 calli via Agrobacterium-mediated transformation [29]. RT-qPCR was performed to determine the relative transcript abundance of OsVIPP1 in RNAi transgenic lines. Primers are provided in Supplementary Table S1.

For OE-OsVIPP1 and OE-OsVIPP1-GUS lines, the full-length CDS of *OsVIPP1* was cloned into the corresponding expression vectors. The resulting recombinant constructs were transformed into ZH11 calli by Agrobacterium-mediated transformation [29]. Positive transgenic plants were verified by PCR and subjected to expression quantification, Southern blot hybridization and phenotypic analyses. Primers are shown in Supplementary Table S1.

### 4.3. Phylogenetic Tree Construction and Multiple-Sequence Alignment

PspA/IM30/VIPP1 homologous protein sequences were retrieved from the NCBI database. Based on the corresponding homology annotations and taxonomic coverage, 25 representative sequences were selected for phylogenetic analysis to ensure broad representation of the sampled taxa. These sequences included representatives of bacteria, cyanobacteria, green algae, bryophytes and angiosperms. The accession numbers of the sequences used in the final analysis are provided in Supplementary Table S3.

Multiple-sequence alignment was performed using MAFFT v7.490 with the --localpair --maxiterate 1000 options. The initial alignment contained 426 amino acid positions and was subsequently trimmed using trimAl v1.5.rev0 with the -strictplus option, resulting in 257 positions being retained for phylogenetic analysis. Substitution-model selection was performed using ModelFinder implemented in IQ-TREE v3.1.2, and the best-fitting model was determined to be Q.INSECT+F+I+G4 according to the Bayesian information criterion (BIC). A maximum-likelihood phylogenetic tree was then constructed using IQ-TREE v3.1.2, with 1000 ultrafast bootstrap replicates and 1000 SH-aLRT replicates used to assess node support. The resulting tree was annotated and graphically refined using the online iTOL tool. Multiple-sequence alignment inspection and visualization were performed using Jalview 2.11.4.0.

### 4.4. Subcellular Localization of OsVIPP1

The full-length CDS of *OsVIPP1* without the stop codon was cloned into the PAN580 vector to generate the 35S::OsVIPP1-GFP fusion expression construct. The recombinant plasmid was introduced into rice protoplasts using MMG solution containing 0.6 M mannitol, 15 mM MgCl_2_, and 4 mM MES (pH 5.7). W5 solution contained 154 mM NaCl, 125 mM CaCl_2_, 5 mM KCl, and 2 mM MES (pH 5.7). After transfection, the protoplasts were incubated in darkness for 12–16 h.

For the osmotic-stress experiment, the transfected protoplasts were treated with H_2_O, MMG, or W5 for 30 min to generate different osmotic pressures. Chloroplast membrane status and OsVIPP1-GFP fluorescence signals were compared among the treatments. Three biological replicates were performed using independently transformed protoplast preparations. Fluorescence signals were observed using a Zeiss LSM980 super-resolution microscope equipped with an Airyscan2 module(ZEISS Microscopy, Oberkochen, Germany).

### 4.5. Observation of Leaf Chloroplast Ultrastructure by Transmission Electron Microscopy

Leaves from WT, *osvipp1* knockout mutants, and OE-OsVIPP1 overexpression lines were harvested at the three-leaf stage. Approximately 1 cm of the leaf tip was removed, and the subsequent approximately 5 mm leaf region was collected, ensuring that all samples were taken from the same leaf position. The leaf tissues were cut into approximately 1 mm × 1 mm pieces, immediately fixed in 2.5% glutaraldehyde, and subjected to vacuum infiltration. After rinsing with 0.1 M PBS, the samples were post-fixed with 1% osmium tetroxide, followed by dehydration through a graded acetone series, infiltration with Epon 812 resin, and embedding. Ultrathin sections (60–80 nm thick) were prepared using a Leica UC6 ultramicrotome equipped with a diamond knife. The sections were stained with 0.5% aqueous uranyl acetate for 30 min, followed by lead citrate staining for 10 min. Chloroplasts in mesophyll cells were examined using an H-7650(Hitachi High-Tech Corporation, Tokyo, Japan) transmission electron microscope operated at 120 kV at the Electron Microscopy Platform of Huazhong Agricultural University. Images were acquired at 5000× magnification using a CCD imaging system. Three independent plants were analyzed, with 10 chloroplasts examined per plant.

### 4.6. qRT-PCR

Total RNA was isolated from leaves of WT and *osvipp1* mutant at the three-leaf stage using TRIzol reagent (TransGene Biotech, Beijing, China). After DNase digestion, cDNA was synthesized by reverse transcription with M-MLV reverse transcriptase (Invitrogen, Beijing, China) and random primers. qRT-PCR was performed on a ViiA™ 7 Real-Time PCR System (Thermo Fisher Scientific, Foster City, CA, USA). with 384-Well Block. The thermal profile consisted of an initial denaturation at 95 °C for 10 min, followed by 40 cycles of 95 °C for 15 s and 60 °C for 1 min. Actin was used as the reference gene. The relative expression level was calculated by the 2^−ΔΔCt^ method [30]. Primers for qRT-PCR are listed in Supplementary Table S4.

### 4.7. Southern Blot Analysis of Transgene Copy Number

Genomic DNA was extracted from three-leaf-stage leaves of WT and OE-OsVIPP1 plants using the CTAB method. Genomic DNA was digested with KpnI and separated by electrophoresis on 0.8% agarose gel, followed by blotting onto a nylon membrane. A 570 bp DIG-labeled probe was generated using the *HPT* fragment as a template for membrane hybridization. After hybridization, the membrane was subjected to sequential washing and immunological color-development. Transgene copy number was determined based on the chemiluminescent imaging signals.

### 4.8. Seed Germination Assay

Seeds of WT, OE-OsVIPP1 and RNAi-OsVIPP1 plants used for the field experiments were harvested in mid-September 2024, when seeds of all three materials had reached full maturity. After harvest, seeds from all materials were dried under the same high-temperature drying conditions for three days and stored at −20 °C. The germination assay was conducted one month after harvest.

Initial seed moisture content, seed-mass distribution and dormancy status were not separately determined in this study. To minimize the potential effects of these factors, all materials were grown under the same field conditions, harvested at comparable maturity stages, and subjected to the same drying and storage procedures. Seeds of comparable size and fullness were randomly selected from each material before the assay.

For surface sterilization, seeds were sequentially treated with 75% ethanol for 1 min and 0.15% HgCl_2_ solution for 15 min, followed by ten washes with sterile distilled water, 1 min per wash. The sterilized seeds were evenly placed on Petri dishes containing moist filter paper and incubated in darkness at 28 °C in a constant-temperature incubator. Fifty seeds were placed in each Petri dish, and three independent Petri dishes were used for each material. Each Petri dish was treated as one experimental unit. The seeds were cultured under identical conditions, and germination was recorded once every 24 h at a fixed time for seven consecutive days. Germination was defined as radicle protrusion through the seed coat. The cumulative number of germinated seeds was recorded at each time point, and the germination percentage was calculated to generate germination dynamics curves.

To quantify the germination rate of different materials, the time required to reach 50% germination (T_50_) was calculated from the cumulative germination data for each Petri dish. T_50_ was defined as the time required to reach 50% of the final total number of germinated seeds and was calculated using the following formula:

T_50_ = t_i_ + [(N/2 − N_i_)(t_j_ − t_i_)]/(N_j_ − N_i_)

where N is the final total number of germinated seeds; N_i_ and N_j_ are the cumulative numbers of germinated seeds at the two consecutive observation time points immediately before and after 50% germination, respectively; and t_i_ and t_j_ are the corresponding observation times. Mean germination time (MGT) was calculated from the germination data for each Petri dish using the following formula:

MGT = Σ(n_i_t_i_)/Σn_i_

where n_i_ is the number of seeds germinated at time t_i_. The mean T_50_ and MGT values were then calculated from three independent Petri dishes and are presented as the mean ± standard deviation (mean ± SD).

### 4.9. GUS Histochemical Staining Assay

Stem tissues from transgenic lines were subjected to GUS histochemical staining. Briefly, samples were immersed in 90% acetone and incubated on ice for 20–30 min with vacuum infiltration for 10 min. Acetone solution was discarded, and stems were rinsed with phosphate buffer. Samples were submerged in GUS staining solution and incubated overnight at 37 °C in the dark. After removal of staining solution, samples were decolorized in 70% ethanol prior to observation and photography.

### 4.10. Investigation of Agronomic Traits

T_2_ plants of WT, RNAi-OsVIPP1 and OE-OsVIPP1 were grown under identical field-management conditions. For each family in each plot, plants were arranged in three rows, with 10 plants per row and 30 plants in total. To minimize border effects, plants 1–3 and 8–10 at both ends of each row were excluded, and plants 4–7 in the middle of each row were retained. Thus, 12 central plants per family in each plot were used for agronomic-trait and grain-related measurements.

After the plants reached full maturity, plant height, effective panicle number per plant, panicle length, 1000-grain weight and grain yield per plant were measured. Plant height was defined as the vertical distance from the soil surface to the tip of the highest panicle. An effective panicle was defined as a panicle bearing at least five filled grains. Panicle length was measured from the base of the main panicle to its tip. The 1000-grain weight was determined by weighing 1000 randomly selected plump grains. Grain yield per plant was defined as the total weight of clean grains obtained after threshing, air-drying and removing unfilled grains and impurities.

### 4.11. Transcriptome Sequencing and Analysis

Transcriptome analysis was performed using mature T_2_ seeds from WT, OE-OsVIPP1 and RNAi-OsVIPP1 plants. Three independent biological replicates were included for each group, resulting in nine sequencing samples in total. Each biological replicate consisted of a pooled sample containing 3 g of uniformly sized mature seeds, which were dehulled before sampling. Because the samples were collected according to mass rather than a fixed number of seeds, each pooled sample was treated as one biological replicate. Total RNA was extracted using TRIzol, and its quality and integrity were assessed using a NanoDrop 2000 spectrophotometer (Thermo Fisher Scientific, Waltham, MA, USA) and an Agilent 2100 Bioanalyzer (Agilent Technologies, Santa Clara, CA, USA). Samples with an RIN of ≥7.0 and a 28S/18S ratio of ≥1.0 were used for mRNA library construction. Messenger RNA was enriched using Oligo (dT) magnetic beads (Thermo Fisher Scientific, Waltham, MA, USA), and paired-end 150 bp sequencing (PE150) was performed on an Illumina platform. The Clean Data output was at least 9.81 Gb per sample, and the Q30 base proportion was at least 95.40% for all samples.

Raw reads were quality-filtered using fastp v0.23.2 by removing reads containing adapter sequences, reads with more than 10% ambiguous bases (N), and reads in which more than 50% of the bases had a quality score of Q ≤ 10. The filtered Clean reads were aligned to the *Oryza sativa* MSU v7.0 reference genome using HISAT2 v2.2.1, with genome alignment rates ranging from 63.76% to 98.28% across samples. Transcript assembly and gene-level read counting were performed using StringTie v2.2.1 with the corresponding MSU v7.0 gene-structure annotation.

FPKM values were used only for gene-expression presentation and sample-correlation analysis. Differential-expression analysis was performed using the unnormalized gene-level raw integer Count matrix generated through the StringTie-based workflow, rather than FPKM values, with DESeq2 v1.42.0. Differentially expressed genes were identified using the criteria of an absolute log2 fold change of ≥1 and an adjusted *p* value (FDR) <0.05. GO and KEGG enrichment analyses were performed using the clusterProfiler R 4.1.1 package based on the hypergeometric test. Multiple-testing-adjusted *p* values, reported as q-values, were used to evaluate the significance of enriched GO terms and KEGG pathways. Enriched terms and pathways were ranked according to their q-values, with lower q-values indicating stronger enrichment significance.

### 4.12. Untargeted Metabolomic Analysis

Untargeted metabolomic profiling was performed on mature rice seeds using a Waters Acquity I-Class PLUS UPLC coupled to a Waters Xevo G2-XS QTOF mass spectrometer(Waters Corporation, Milford, MA, USA). Three independent biological replicates were analyzed for each of WT, OE-OsVIPP1 and RNAi-OsVIPP1. Each replicate consisted of 30 uniformly developed, dehulled ZH11 seeds. The seeds were ground in liquid nitrogen at 60 Hz for 2 min, and 50 mg of powder was extracted with 500 μL of methanol/acetonitrile/water (2:2:1, *v*/*v*/*v*) containing L-2-chlorophenylalanine (2 μg/mL) as an internal standard. Samples were sonicated at 250 W and 4 °C for 10 min, centrifuged at 12,000× *g* and 4 °C for 15 min, vacuum-dried, reconstituted in 100 μL of acetonitrile/water (1:1, *v*/*v*), and centrifuged again under the same conditions for 10 min.

Chromatographic separation was performed on a Waters HSS T3 C18 column (100 mm × 2.1 mm, 1.8 μm) at 40 °C, with a flow rate of 0.4 mL/min and an injection volume of 2 μL. Data were acquired in ESI+ and ESI− modes. The mobile phases and gradient were as follows: 0.1% formic acid in water/acetonitrile for ESI+, 5 mM ammonium acetate in water/acetonitrile for ESI-, 5% B for 0–1 min, 5–95% B for 1–9 min, 95% B for 9–12 min, 95–5% B for 12–12.1 min, and 5% B for 12.1–15 min. MSE data were acquired over m/z 50–1200 using collision energies of 6 eV and 20–40 eV. Leucine-enkephalin was used for lock-mass correction, with a mass error within 5 ppm.

Raw data were collected using MassLynx v4.2 and processed using Progenesis QI v2.4. Pooled QC and reagent-blank samples were included, and samples were injected in randomized order with one QC and one blank after every 10 injections. Features detected in at least 80% of QC samples and in at least 50% of samples within each group were retained. Features associated with blanks or with QC peak-area RSD >30% were removed, and missing values were replaced by one-half of the minimum detected value. Data were normalized using the internal-standard and total-peak areas and corrected using QC-based robust spline correction. Metabolites were annotated using METLIN, KEGG v107, HMDB v4.0, LipidMaps v2021 and an in-house standard library. The identification levels were MSI Level 2, with some metabolites confirmed at MSI Level 1.

PCA was used to assess overall metabolic variation. OPLS-DA models were constructed using the ropls R package and evaluated by 7-fold cross-validation and 200 permutation tests. Differential metabolites were identified using two-sided independent-samples t-tests with Benjamini–Hochberg correction. Metabolites with an absolute log2 fold change ≥ 1, adjusted FDR < 0.05 and VIP ≥ 1 were considered differential metabolites.

### 4.13. Combined Transcriptome–Metabolome Analysis

For the joint transcriptome–metabolome analysis, fold changes were calculated as the abundance in OE-OsVIPP1 divided by that in WT for the OE-OsVIPP1 vs. WT comparison, and as the abundance in RNAi-OsVIPP1 divided by that in WT for the RNAi-OsVIPP1 vs. WT comparison. Up-regulated genes in the OE-OsVIPP1 vs. WT comparison and down-regulated genes in the RNAi-OsVIPP1 vs. WT comparison were selected using an FC threshold of 1.5 and an FDR threshold of 0.01. The intersection of these two gene sets was defined as the target gene set, corresponding to the expected pattern of OE-OsVIPP1 > WT > RNAi-OsVIPP1.

For metabolites, differential features were selected using the criteria of an absolute log2 fold change of ≥1, an adjusted FDR < 0.05 and an OPLS-DA VIP value of ≥1. The intersection of metabolites with higher abundance in OE-OsVIPP1 than in WT and lower abundance in RNAi-OsVIPP1 than in WT was defined as the target metabolite set. The corresponding gene-expression and metabolite-abundance data were integrated into a joint feature matrix and subjected to principal component analysis (PCA) in R. The PCA results were visualized using the ggplot2 package.

### 4.14. Determination of Phytohormone, Soluble Sugar and Fatty Acid Content

Targeted metabolomic analysis was performed on WT, OE-OsVIPP1 and RNAi-OsVIPP1 samples, with three independent biological replicates per group.

The 27 phytohormones were extracted using an isopropanol/water solvent containing isotope-labeled internal standards and analyzed in multiple-reaction monitoring mode on a Waters ACQUITY I-Class UPLC coupled to a SCIEX QTRAP 6500+ mass spectrometer. Calibration curves were generated from the peak-area ratios of each phytohormone to its corresponding isotope-labeled internal standard using weighted 1/x least-squares regression. The final phytohormone contents were calculated based on the measured concentration, final volume, concentration factor and sample mass.

The 37 fatty acids were extracted and converted to fatty acid methyl esters by acid-catalyzed methylation, followed by analysis using an Agilent 7820 GC coupled to an Agilent 5977 mass spectrometer in selected-ion-monitoring mode. Fatty acids were identified by comparing their retention times and mass spectra with those of authentic standards and quantified using external calibration curves prepared from mixed standards. The final fatty acid contents were calculated based on the calibrated concentration, extraction volume, dilution factor and sample mass.

The 13 soluble sugars were extracted with 80% ethanol and analyzed using a Thermo ICS 5000+ ion chromatography system (Thermo Fisher Scientific, Sunnyvale, CA, USA). Sugars were identified by comparison with authentic standards and quantified using external calibration curves. Metabolites with an absolute log_2_ fold change ≥1, adjusted FDR < 0.05 and VIP ≥ 1 were considered differential metabolites. Pairwise comparisons were performed using Student’s *t*-tests, and data analysis and visualization were conducted using GraphPad Prism 9.

In total, 27 phytohormones, 13 soluble sugars and 37 fatty acids were quantified. Detailed information on the individual compounds is provided in Supplementary Tables S5–S7.

### 4.15. Statistical Analysis

All experiments were performed with at least three biological replicates. Data are presented as mean ± SD. Student’s *t*-test was used for comparisons between two groups. For multiple-group comparisons, one-way ANOVA followed by post hoc multiple comparison test was applied. *p* < 0.05 was considered statistically significant; * and ** denote *p* < 0.05 and *p* < 0.01, respectively. For transgenic materials, each independent transformation event was treated as one biological replicate.

## Data Availability

The RNA-seq datasets generated in this study will be deposited in the NCBI Sequence Read Archive (SRA) database, and the metabolomics datasets will be deposited in the MetaboLights database upon acceptance of this manuscript. The accession numbers will be provided after data submission.

## Supplementary Materials

The following supporting information can be downloaded at: https://www.mdpi.com/article/10.3390/plants15192946/s1, Supplementary Figure S1. Subcellular localization of OsVIPP1 and transmission electron microscopy analysis of chloroplast ultrastructure. Supplementary Figure S2. Effect of osmotic stress on the subcellular distribution of OsVIPP1. Scale bar = 3 μm. Supplementary Figure S3. Expression changes of chloroplast genes in the *osvipp1* mutant. Supplementary Figure S4. GUS staining of stems. Supplementary Figure S5. Principal component analysis (PCA) and Venn diagram analyses of transcriptomic and metabolomic data. Supplementary Figure S6. Genotypic identification of OsVIPP1-knockout calli from two independent T_0_ families. Supplementary Figure S7. Schematic map of the pCR8/GW/TOPO entry vector. Supplementary Figure S8. Schematic map of the DS355 destination vector. Supplementary Figure S9. Schematic map of the complementation construct pCAMBIA1300. Supplementary Figure S10. Southern blot analysis to determine the copy number of exogenous inserts in transgenic lines. Supplementary Figure S11. Transcript levels of *OsVIPP1* in overexpression lines. Supplementary Figure S12. Transcript levels of *OsVIPP1* in RNAi lines. Supplementary Table S1. List of primers used for the construction of vectors and mutants. Supplementary Table S2. Information on the *OsVIPP1* sgRNA target sites. Supplementary Table S3. The accession numbers of the sequences used for phylogenetic-tree construction. Supplementary Table S4. List of primers used for qRT-PCR analysis of chloroplast genes. Supplementary Table S5. List of plant hormones analyzed for quantification. Supplementary Table S6. List of soluble sugars analyzed for quantification. Supplementary Table S7. List of fatty acids analyzed for content determination. Supplementary Table S8. Time required for different materials to reach 50% germination and complete germination.
