# Supplementary material for: OsVIPP1 Supports Rice Leaf Chloroplast Development and Is Associated with Seed Metabolic Changes and Yield-Related Traits
Source: Plants (Basel). 2026 Sep 26;15(19):2946. doi: 10.3390/plants15192946 (PMC13666959; doi:10.3390/plants15192946)
Supplement: Supplementary file 1 [file plants-15-02946-s001.zip › plants-4544711-supplementary.pdf]

Supporting information

Supplementary Figures

Supplementary Figure S1. Subcellular localization of OsVIPP1 and transmission electron microscopy analysis of chloroplast ultrastructure.

Supplementary Figure S2. Effect of osmotic stress on the subcellular distribution of OsVIPP1. Scale bar = 3 μm.

Supplementary Figure S3. Expression changes of chloroplast genes in the *osvipp1* mutant.

Supplementary Figure S4. GUS staining of stems. Scale bar = 3 cm.

Supplementary Figure S5. Principal component analysis (PCA) and Venn diagram analyses of transcriptomic and metabolomic data.

Supplementary Figure S6. Genotypic identification of OsVIPP1-knockout calli from two independent T_0 families.

Supplementary Figure S7. Schematic map of the pCR8/GW/TOPO entry vector.

Supplementary Figure S8. Schematic map of the DS355 destination vector.

Supplementary Figure S9. Schematic map of the complementation construct pCAMBIA1300.

Supplementary Figure S10. Southern blot analysis to determine the copy number of exogenous inserts in transgenic lines.

Supplementary Figure S11. Transcript levels of *OsVIPP1* in overexpression lines.

Supplementary Figure S12. Transcript levels of *OsVIPP1* in RNAi lines.

Supplementary Tables

Supplementary Table S1. List of primers used for the construction of vectors and mutants.

Supplementary Table S2. Information on the *OsVIPP1* sgRNA target sites.

Supplementary Table S3. The accession numbers of the sequences used for phylogenetic-tree construction.

Supplementary Table S4. List of primers used for qRT-PCR analysis of chloroplast genes.

Supplementary Table S5. List of plant hormones analyzed for quantification.

Supplementary Table S6. List of soluble sugars analyzed for quantification.

Supplementary Table S7. List of fatty acids analyzed for content determination.

Supplementary Table S8. Time required for different materials to reach 50% germination and complete germination.

a

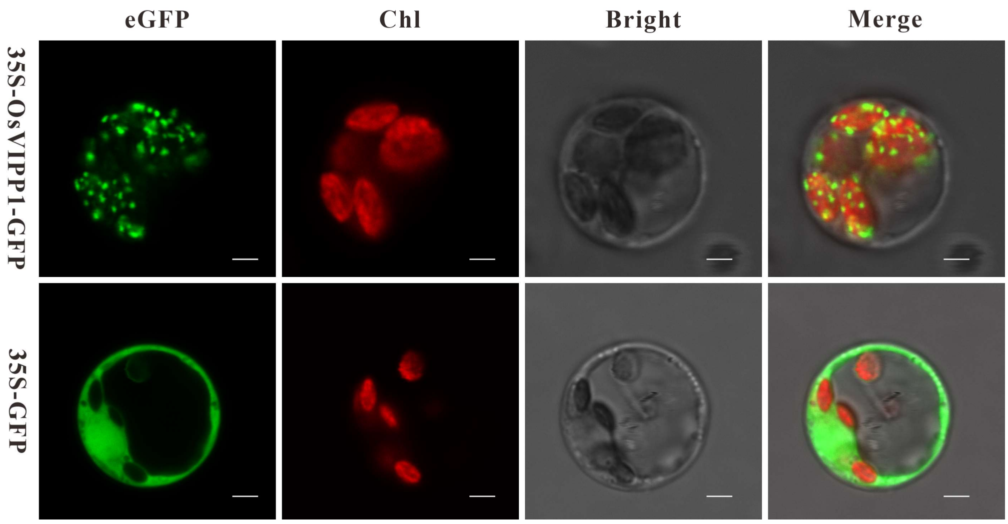

b

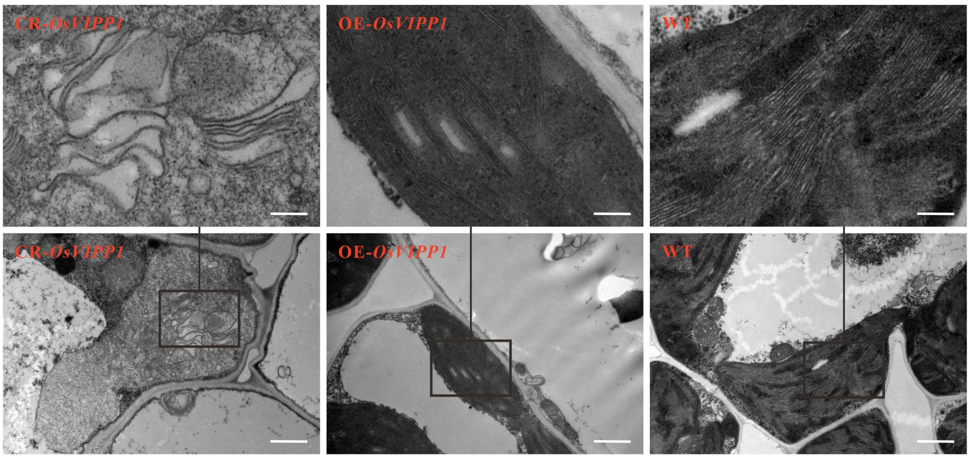

Supplementary Figure S1. Subcellular localization of OsVIPP1 and transmission electron microscopy analysis of chloroplast ultrastructure. (a) Subcellular localization of 35S::OsVIPP1-GFP in rice protoplasts compared with the 35S::GFP empty vector control. GFP fluorescence, chloroplast autofluorescence, bright-field images, and merged images are shown. Scale bar = 3 μm. (b) Transmission electron microscopy analysis of leaves from WT, the *osvipp1* mutant, and OsVIPP1-overexpressing plants (OE-OsVIPP1). Scale bars = 0.4 μm and 1 μm.

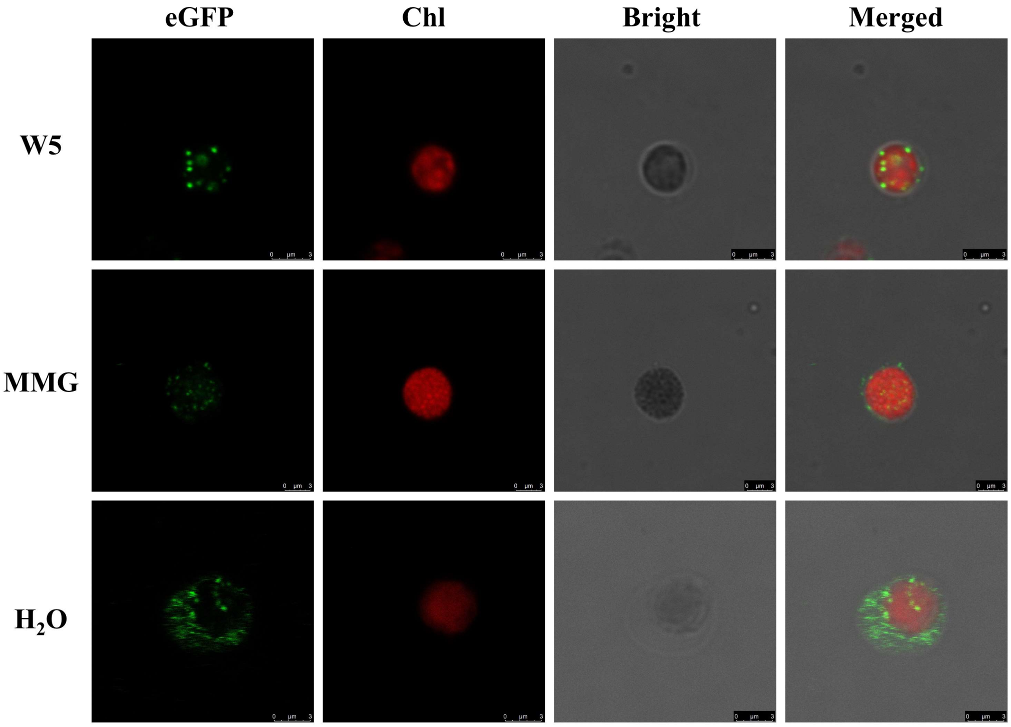

Supplementary Figure S2. Effect of osmotic stress on the subcellular distribution of OsVIPP1. Scale bar = 3 μm.

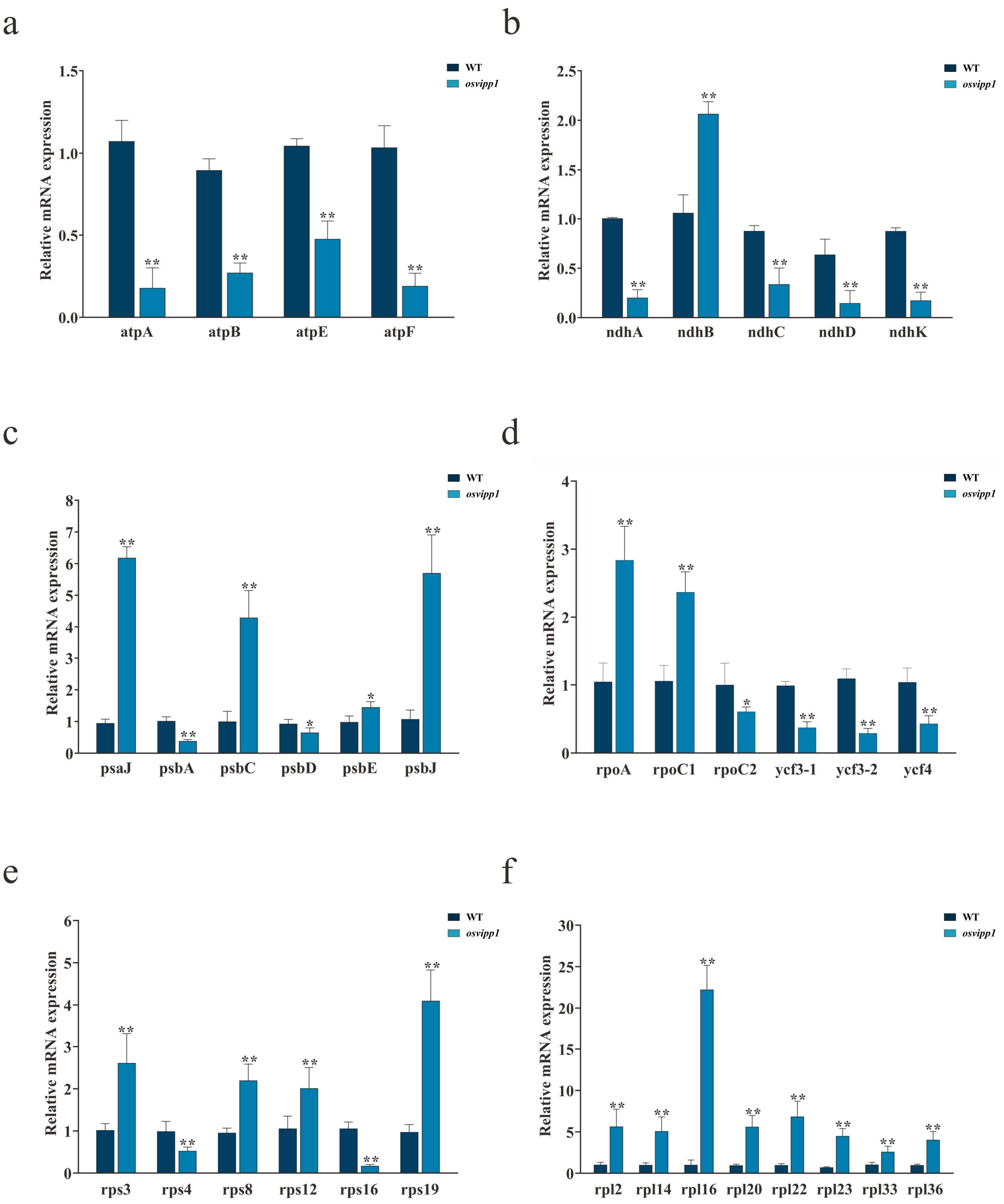

Supplementary Figure S3. Expression changes of chloroplast genes in the *osvipp1* mutant. (a–f) qRT-PCR analysis of chloroplast-related genes in WT and the *osvipp1* mutant, including genes associated with ATP synthase, the NADH dehydrogenase-like (NDH) complex, photosystem I, photosystem II, chloroplast transcription, and chloroplast translation-related ribosomal proteins.

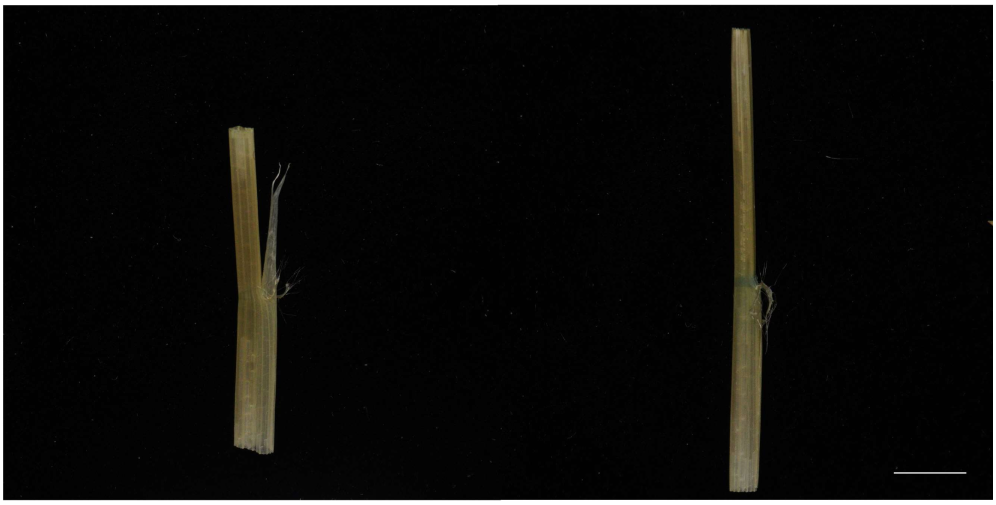

WT OE-OsVIPP1-GUS

Supplementary Figure S4. GUS staining of stems. Scale bar = 3 cm.

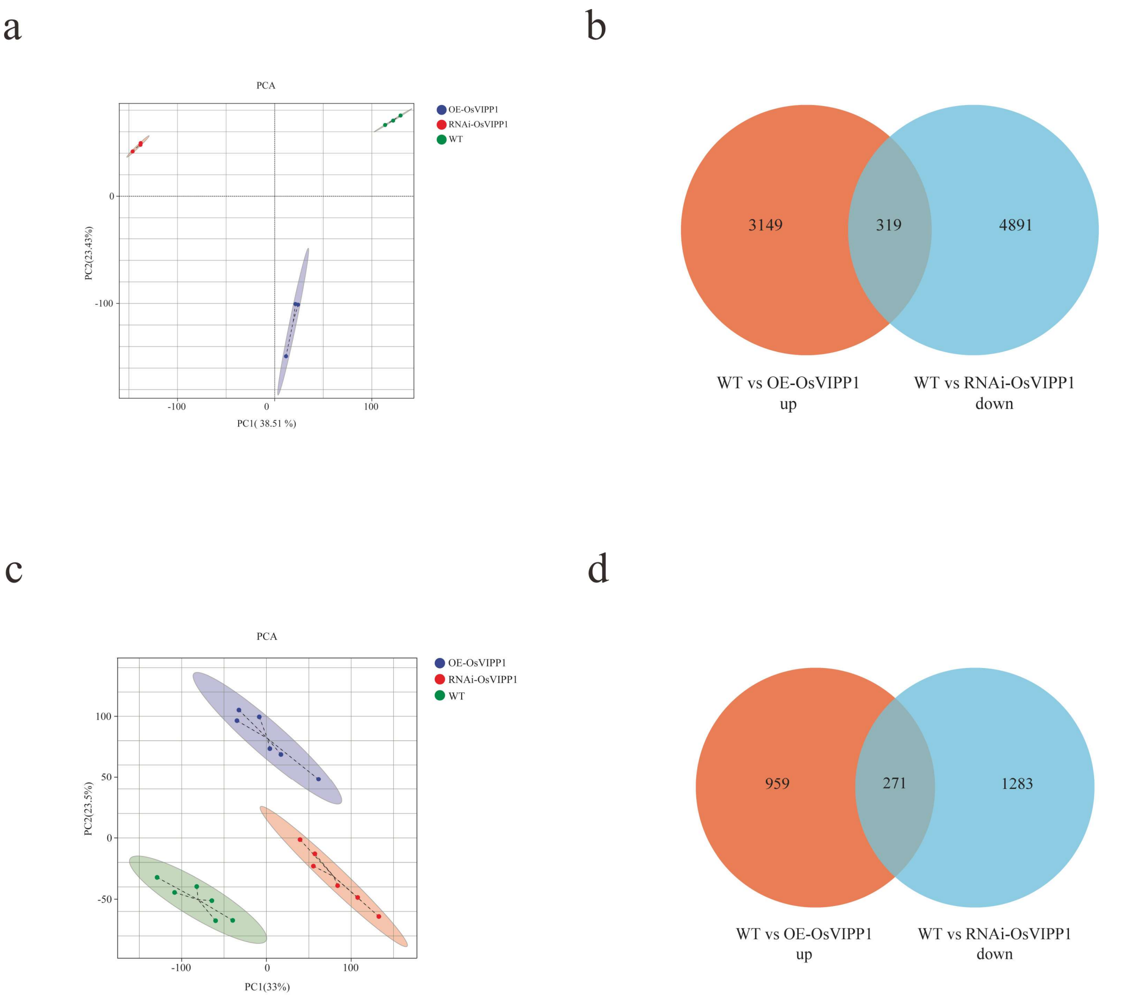

Supplementary Figure S5. Principal component analysis (PCA) and Venn diagram analyses of transcriptomic and metabolomic data. (a) PCA analysis of transcriptomic data from WT, OE-OsVIPP1, and RNAi-OsVIPP1 plants. (b) Venn diagram analysis of common upregulated genes in WT vs OE-OsVIPP1 and downregulated genes in WT vs RNAi-OsVIPP1. (c) PCA analysis of metabolomic data from WT, OE-OsVIPP1, and RNAi-OsVIPP1 plants. (d) Venn diagram analysis of common upregulated metabolites in WT vs OE-OsVIPP1 and downregulated metabolites in WT vs RNAi-OsVIPP1.

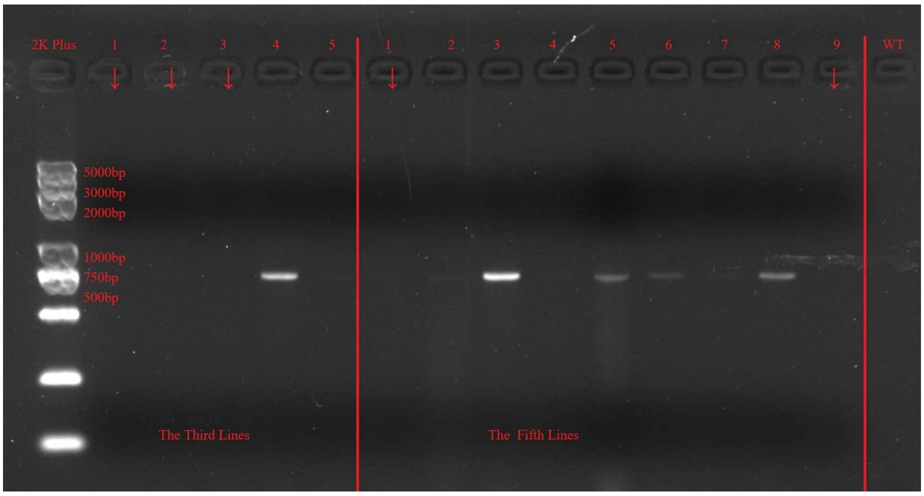

Supplementary Figure S6. Genotypic identification of OsVIPP1-knockout calli from two independent T_0 families. A total of five OsVIPP1 complete-knockout calli were identified in Family 3, among which three were *HPT*-negative. Nine OsVIPP1 complete-knockout calli were obtained in Family 5, including two HPT-negative individuals.

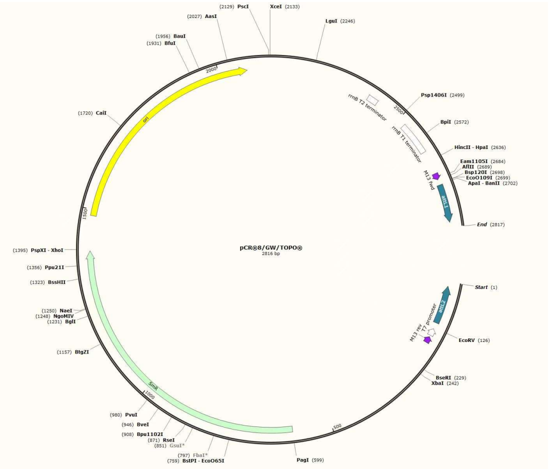

Supplementary Figure S7. Schematic map of the pCR8/GW/TOPO entry vector.

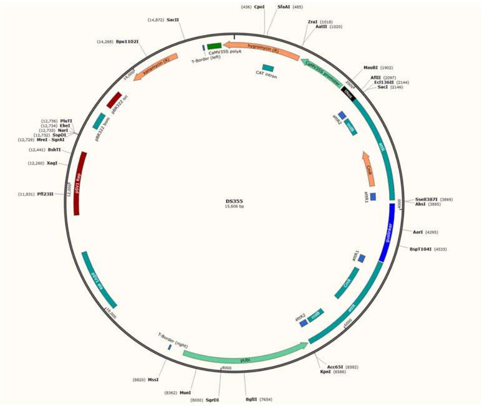

Supplementary Figure S8. Schematic map of the DS355 destination vector.

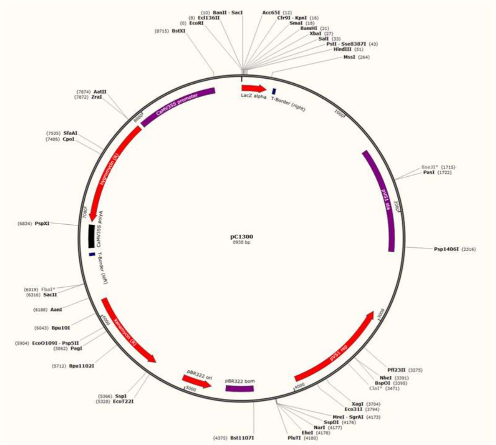

Supplementary Figure S9. Schematic map of the complementation construct pCAMBIA1300.

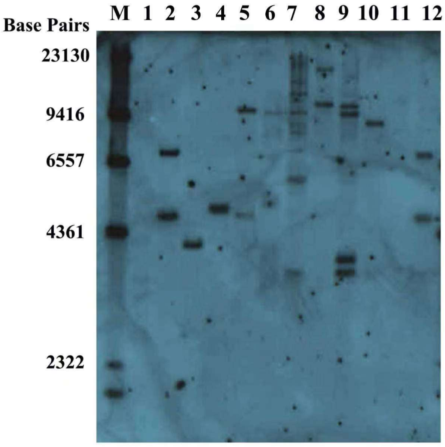

Supplementary Figure S10. Southern blot analysis to determine the copy number of exogenous inserts in transgenic lines. Lane 1: wild type (WT); the remaining lanes represent positive *OsVIPP1* overexpression lines.

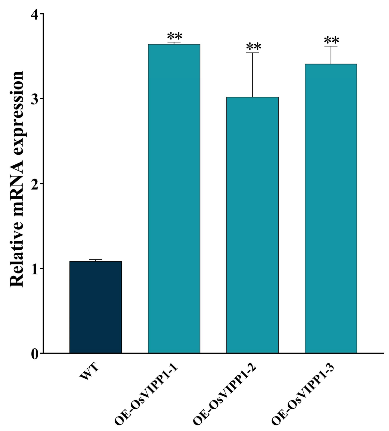

Supplementary Figure S11. Transcript levels of *OsVIPP1* in overexpression lines.

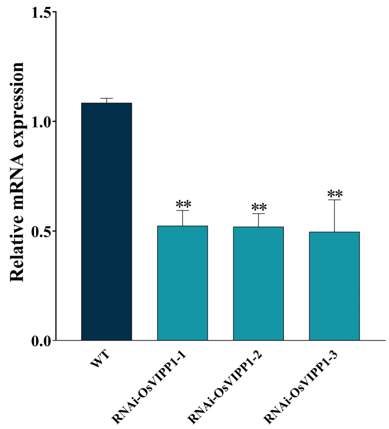

Supplementary Figure S12. Transcript levels of *OsVIPP1* in RNAi lines.

Supplementary Table S1. List of primers used for the construction of vectors and mutants.

Name	Primer sequence (F)	Primer sequence (R)	Purpose
CR-VIPP1-1	ggcAGAATGAGCTAAATGAGCTG	aaacCAGCTCATTTAGCTCATTC	CRISPR-mediated gene knockout
CR-VIPP1-2	gccGCTTGATCAGCAAAAGGGCG	aaacCGCCCTTTTGCTGATCAAG	CRISPR-mediated gene knockout
VIPP1	TCGGAGGGCTCAACTTGCTC	AAGGCTGCACTGGTGGTTCA	Identification of gene knockout mutant
Pro-VIPP1-ter	gacgtagtggtggtaccagt	caaaagaacaaaaaaaatca	PCR amplification of the promoter-OsVIPP1-terminator
PC-VIPP1	atttggcccacattcttccgATGGAGATTAG GGCGCCGCCGA	tcggcggcgccctaatctccatCGGAAGAAT GTGGGCCAAAT	PCR amplification of the promoter and CDS
PCG-VIPP1	gatctacagcgctgaagcttgg TGGGTTAGATTAGAGGAAGA	acataagggactgaccacccgGGATATTCAT TGGCCTTCTTCC	Construction of the GUS vector
RNAi-VIPP1	acttagacttattcaagtgc	ctggtctaggatcttttcag	RNA interference
PA-VIPP1	taagtccggagctagctctagaATGGAGATT AGGGCGCCGCCGA	cgcccttgctcaccatggatccTATTCATTGG CCTTCTTCCG	subcellular localization
OE-VIPP1	aggtcgactctagaggatccATGGAGATTA GGGCGCCGCCGA	taagaaacgagctcggtaccTATTCATTGGC CTTCTTCCG	overexpression
QRT-ACTIN	TGGCATCTCTCAGCACATTCC	TGCACAATGGATGGGTCAGA	gene expression level in tissues and mutant plants
QRT-VIPP1	AAAAGGGCGTGGTTGAGAAC	TACATTCCCCAGCATTTCGC	gene expression level in tissues and mutant plants

Supplementary Table S2. Information on the *OsVIPP1* sgRNA target sites

Target	Genomic coordinates	Strand	Spacer sequence	PAM
target 1	NC_089035.1:39544722-39544744	-	GCTTGATCAGCAAAAGGGCG	TGG
target 2	NC_089035.1:39542875-39542897	-	AGAATGAGCTAAATGAGCTG	CGG

Supplementary Table S3. The accession numbers of the sequences used for phylogenetic-tree construction.

Accession	Domain	Phylum	Genus	Species
NP_388499.1	Bacteria	Bacillota	Bacillus	Bacillus subtilis, strain 168
NP_415820.1	Bacteria	Pseudomonadota	Escherichia	Escherichia coli, strain K-12 MG1655
NP_460648.1	Bacteria	Pseudomonadota	Salmonella	Salmonella enterica serovar Typhimurium, strain LT2
YP_001006361.1	Bacteria	Proteobacteria	Yersinia	Yersinia enterocolitica, strain 8081
WP_005425704.1	Bacteria	Pseudomonadota	Vibrio	Undetermined
WP_010874316.1	Bacteria	Cyanobacteriota	Synechocystis	Undetermined
WP_043695356.1	Bacteria	Cyanobacteriota	Undetermined	Undetermined
WP_028949083.1	Bacteria	Cyanobacteriota	Synechocystis	Undetermined
NP_564846.1	Viridiplantae	Tracheophyta	Arabidopsis	Arabidopsis thaliana
NP_001130281.1	Viridiplantae	Tracheophyta	Zea	Zea mays
NP_001354008.1	Viridiplantae	Tracheophyta	Zea	Zea mays
VAH26830.1	Viridiplantae	Tracheophyta	Triticum	Triticum turgidum subsp. durum
VAH67262.1	Viridiplantae	Tracheophyta	Triticum	Triticum turgidum subsp. durum
NP_001383800.1	Viridiplantae	Tracheophyta	Oryza	Oryza sativa, Japonica Group
KRH24567.1	Viridiplantae	Tracheophyta	Glycine	Glycine max
NP_001242008.2	Viridiplantae	Tracheophyta	Glycine	Glycine max
XP_004250100.1	Viridiplantae	Tracheophyta	Solanum	Solanum lycopersicum
XP_003552456.1	Viridiplantae	Tracheophyta	Glycine	Glycine max
XP_024367355.1	Viridiplantae	Bryophyta	Physcomitrium	Physcomitrium patens
XP_001693830.1	Viridiplantae	Chlorophyta	Chlamydomonas	Chlamydomonas reinhardtii
XP_002948865.1	Viridiplantae	Chlorophyta	Volvox	Volvox carteri f. nagariensis
XP_002949072.1	Viridiplantae	Chlorophyta	Volvox	Volvox carteri f. nagariensis
PNW76694.1	Viridiplantae	Chlorophyta	Chlamydomonas	Chlamydomonas reinhardtii
XP_005643904.1	Viridiplantae	Chlorophyta	Coccomyxa	Coccomyxa subellipsoidea, strain C-169
XP_005645023.1	Viridiplantae	Chlorophyta	Coccomyxa	Coccomyxa subellipsoidea, strain C-169

Supplementary Table S4. List of primers used for qRT-PCR analysis of chloroplast genes.

Gene name	Primer sequence (F)	Primer sequence (R)
atpA	TCGCGGAGTTACAAGCCTTT	ATTCTCGTAATCGTCGCCCG
atpB	GGGGAGCTGGAGTAGGTAA	CCCCTACTCCGCCAAATACG
atpE	ACTGACTCCTAAGCGAATT	GGCGTGGTTTTGGTAATAC
atpF-ex	AATTGCGTAGAGGAACCATT	TCTCGAGTATCCATTCATTGG
atpF-in	CGATCTCGACGAATTACTTC	GTGTTAATGGGTCTCATATTATTGG
atpH	CGTAGGCTCTTGCTTCTAT	GTCTCGCAATACCTTCTA
atpI	ATTAGTCGTTGTTGTTCTTGT	GCTTGAATACCGGCTTGTAA
cemA	TTTGCCTTGGGGAGTCTCTT	TTGTATTATTTCCCTTGCGTA
clpP	ATGTCAGCAGACGAAGCCAA	TCAGTATCGCAGTGCTCGTC
matK	TGCAATGGTTTGGGGTTCCGGTA	TGTGCGATCCACGAAAGAA
ndhA-ex	GATGCGGATATTTCTCTTTCT	TCTATGGACTGATATGGATTCT
ndhA-in	ACGACGGAAAGAAATAAAATAAGA	AATGGCTATCAAGAACGAATT
ndhB-ex	AAGTCCTACAGTGATGGATAAAG	CGGTCTAATGAGGCTACTATG
ndhB	TACGAAGGATCCCCACTCC	TCATCCTTCAGAGACTACGA
ndhB-in	AATCCTTCATCAGTGGTTGT	TCATCCTTCAGAGACTACGA
ndhC	CCCTTGGGCAATGAGTTTCG	CATTCCAAGGCTCCTTTTCGC
ndhE	TGGATTGATCACAAGCCGAAA	AGCAGCCGCAAGGGGCTATAA
ndhF	CTGCGGGGATTTTTCTTATA	GGCTAAGCTTCTTTTAATAT
ndhG	TCGCTAGGATTAGTTCTTGTT	TTAGACCATTCTGAGCCATT
ndhH	GTCCGACGCTTTAAAGAAA	AAGACTATCATCTCTACCA
ndhI	AATACGCGCAGCAAGGTACA	CCGGAAACGCTCCGATGTAA
ndhJ	TCCGATTTTCAAGAACGCGAAT	GGGCCAGCCTATCCAACTTT
ndhK	TCCTAGGCAAGCGGACCTAA	CAAGCTCCCATAGCAATGACG
petA	GCAGTGCTTCCCGATACTGT	AGCACCCACATTCAAACCCTC
petB	ACTGGAAGTGCTAGTGTGG	CGGAGTAAGCAGAGAAGGT
petD	CCGACGGGCTTATTGACAGT	GGTTGTAGCTACTGGGGCGAC
petL	CTAGTTATTTCGGTTTCTATTGG	GACGTATCTTGTTCCAAGCC
petN	GGATATAGTAAGTCTCGCTTGG	CTAGAGTCCACCTCCCTCC
psaA	GCAGTCGGATGTTTGGGGTA	GAAATCTCGGAGCACCCCAT
psaB	ATGAACGGACACCTTTGGCT	CCCACCAATCTTGCTTGCAC
psbC	GCTCCTTTAGGCTCTTTA	GTCGCTAACCAACTTCTA
psbD	GGCCCAATCGCTGTTTTTGT	CCAAAACTCGGCGCAAAGAA
psbE	CCCTATTCATTGCGGGTTGG	ACGGAATTCCTTGTGCGGTT
psbF	TTTTACAGTGCGATGGCTGG	CGTTGGATGAACTGCATTGCT
psbJ	TGGCCGATACTACTGGAAGA	CCCAATCCAGAATATGAACCGT
psbK	TTTCCTTCGCCCAAATTGCCCG	CAGCTTGCCAAACAAAAGGCT
psbL	ATGACACAATCAAACCCGAATG	ACAAAAATGAGTAATAAACCCAGT
psbM	ATATTCTCGCATTTATTGCTACTG	AATCATTTTTGGCTGACTGTTTT
psbN	GGAAACAGCAACTTTAGTCG	CGTGTTCTTCGAATGGATGCT

psbZ	GCTTCTCCTGATGGTTGGTTCA	AGCTACCAGAAAGACCAGTCC
rbcL	AGGGGAACGCGGAAATGACTT	AAAGATACCGCGAGCACGAT
rpl2-ex	ACGATTCTACCAGATATGTCTT	GGAACCATAGATAGGCAAGT
rpl2-in	CGTTCCGTCATCTGGCTTAT	TGATGGTCAGAGGCGGAATTG
rpl14	CAGTGCCCCAAATGCCTCTA	GCTGCATTGTGCGTCATAGCG
rpl16	ACGTATGGGTTCGGGAAAGG	CGGATACTCCGCCCATTCA
rpl22	TGGATGAGATTCGTTGGCGT	GAGTAGCATTTGCTGCTGCC
rpl23	AGCATTGGGGTCGAACTCTTCT	AGGGCCCATTCTTCTACCCCT
rpl36	TACAAAATGTGCTGATTGCGTAGG	ACCCCTGTCTTTTGTTATGCTTC
rpoA	CCGCCTTCCGTAGAAATCGT	CGCGATCTCTCTTGATCCGT
rpoB	GGCAGAAGAACTTTGAGAA	GGAAGAATAGGTGTGATTGATT
rpoC1	CGTGCTACTTCTAATGTT	CCTGTATGCCTAATCTATG
rpoC2	GGCTGATGATGAGGTAAG	TGAAGAATAGAAGGATAGACAA
rps8	CGACCTGGCTTACGAATTTA	ACTTCTCCCCCAAATTCTGTT
rps11	GCTGGTAGTGGGAAGAGAT	CGGCATAGGTGTTTACATC
rps12-ex	TTGCCAGAGTACGATTAACCT	TCACTCAAACTCCAAAGGTTAAACT
rps12-in	ATGTGAAGTGTATGGCTAACC	GCCCTTGTTGACGATTCTTT
rps14	GAGAGGAAGCGGCAGAAAT	CACGGACAAAGTAAGGGGAAA
ycf4	ATTATATCTGTACTTCCATCTCA	GTCATAACCACTGCCTAC

Supplementary Table S5. List of plant hormones analyzed for quantification.

Number	Name	Abbreviation
1	1-Aminocyclopropanecarboxylic acid	ACC
2	Indole-3-acetic acid	IAA
3	Methyl indole-3-acetate	MeIAA
4	3-Indolebutyric acid	IBA
5	N6-Isopentenyladenine	iP
6	Kinetin	KT
7	trans-Zeatin	tZ
8	Cis-Zeatin	cZ
9	Dihydrozeatin	DHZ
10	Methyl jasmonate	MeJA
11	Isopentenyl adenosine	iPA
12	trans-Zeatin-riboside	tZR
13	Brassinolide	BL
14	Melatonine	MT
15	Strigolactone	SL
16	Salicylic acid	SA
17	Indole-3-carboxaldehyde	ICA
18	(±)-Jasmonic acid	JA
19	Dihydrojasmonic acid	DHJA
20	Abscisic acid	ABA
21	Gibberellin A7	GA7
22	Gibberellin A4	GA4
23	Gibberellin A1	GA1
24	Methylsalicylate	MeSA
25	Gibberellin A3	GA3
26	trans-Indole-3-acrylic Acid	I3A
27	3-Indole propionic acid	IPA

Supplementary Table S6. List of soluble sugars analyzed for quantification.

Number	Name	Abbreviation
1	Glucose	Glc
2	Fructose	Fru
3	Trehalose	Tre
4	Maltose	Mal
5	Sucrose	Suc
6	Fucose	Fuc
7	Rhamnose	Rha
8	Arabinose	Ara
9	Galactose	Gal
10	Lactose	Lac
11	Stachyose	Sta
12	Raffinose	Raf
13	Mannose	Man

Supplementary Table S7. List of fatty acids analyzed for content determination.

Number	Name	Abbreviation
1	Butyric acid	C4:0
2	Hexanoic acid	C6:0
3	Octanoic acid	C8:0
4	Decanoic acid	C10:0
5	Undecanoic acid	C11:0
6	Lauric acid	C12:0
7	Tridecanoic acid	C13:0
8	Myristic acid	C14:0
9	Myristoleic acid	C14:1
10	Pentadecanoic acid	C15:0
11	cis-10-Pentadecenoic acid	C15:1
12	Palmitic acid	C16:0
13	Palmitoleic acid	C16:1
14	Heptadecanoic acid	C17:0
15	cis-10-heptadecenoic acid	C17:1
16	Stearic acid	C18:0
17	Elaidic acid	C18:1n9t
18	Oleic acid	C18:1n9c
19	Linolelaidic acid	C18:2n6t
20	Linoleic acid	C18:2n6c
21	Arachidic acid	C20:0
22	Gamma-Linolenic acid	C18:3n6
23	11-Eicosenoic acid	C20:1
24	Linolenic acid	C18:3n3
25	Henicosanoic acid	C21:0
26	11C,14C-Eicosadienoic acid	C20:2
27	Docosanoic acid	C22:0
28	cis-8,11,14-Eicosatrienoic acid	C20:3n6
29	Erucic acid	C22:1n9
30	cis-11,14,17-Eicosatrienoic acid	C20:3n3
31	Tricosanoic acid	C23:0

32	Arachidonic acid	C20:4n6
33	13C,16C-Docosadienoic acid	C22:2
34	Lignoceric acid	C24:0
35	Eicosapentaenoic acid	C20:5n3
36	Nervonic acid	C24:1
37	cis-4,7,10,13,16,19-Docosahexaenoic acid	C22:6n3

Supplementary Table S8. Time required for different materials to reach 50% germination and complete germination.

	**T50（day, mean ± SD）**	**MGT（day, mean ± SD）**
WT	2.30 ± 0.04	2.74 ± 0.03
OE-OsVIPP1	3.61 ± 0.05	4.28 ± 0.03
RNAi-OsVIPP1	3.77 ± 0.11	4.40 ± 0.07
